# Shortwave Infrared Imaging Enables High-Contrast Fluorescence-Guided Surgery in Neuroblastoma

**DOI:** 10.1158/0008-5472.CAN-22-2918

**Published:** 2023-03-19

**Authors:** Laura Privitera, Dale J. Waterhouse, Alessandra Preziosi, Irene Paraboschi, Olumide Ogunlade, Chiara Da Pieve, Marta Barisa, Olumide Ogunbiyi, Gregory Weitsman, J. Ciaran Hutchinson, Kate Cross, Lorenzo Biassoni, Danail Stoyanov, Neil Sebire, Paul Beard, Paolo De Coppi, Gabriela Kramer-Marek, John Anderson, Stefano Giuliani

**Affiliations:** 1 University College London, Wellcome/EPSRC Centre for Interventional and Surgical Sciences, London, United Kingdom.; 2 Cancer Section, Developmental Biology and Cancer Programme, UCL Great Ormond Street Institute of Child Health, London, United Kingdom.; 3 Department of Paediatric Surgery, Fondazione IRCCS Ca' Granda Ospedale Maggiore Policlinico di Milano, Milano, Italy.; 4 Department of Paediatric Urology, Fondazione IRCCS Ca' Granda Ospedale Maggiore Policlinico di Milano, Milano, Italy.; 5 University College London, Department of Medical Physics and Biomedical Engineering, London, United Kingdom.; 6 Preclinical Molecular Imaging, Division of Radiotherapy and Imaging, The Institute of Cancer Research, London, United Kingdom.; 7 Department of Histopathology, Great Ormond Street Hospital for Children NHS Trust, London, United Kingdom.; 8 Richard Dimbleby Department of Cancer Research, School of Cancer and Pharmaceutical Sciences, King's College London, Guy's Campus, London, United Kingdom.; 9 Department of Specialist Neonatal and Paediatric Surgery, Great Ormond Street Hospital for Children NHS Trust, London, United Kingdom.; 10 Department of Radiology, Great Ormond Street Hospital for Children NHS Trust, London, United Kingdom.

## Abstract

Fluorescence-guided surgery is set to play a pivotal role in the intraoperative management of pediatric tumors. Shortwave infrared imaging (SWIR) has advantages over conventional near-infrared I (NIR-I) imaging with reduced tissue scattering and autofluorescence. Here, two NIR-I dyes (IRDye800CW and IR12), with long tails emitting in the SWIR range, were conjugated with a clinical-grade anti-GD2 monoclonal antibody (dinutuximab-beta) to compare NIR-I and SWIR imaging for neuroblastoma surgery. A first-of-its-kind multispectral NIR-I/SWIR fluorescence imaging device was constructed to allow an objective comparison between the two imaging windows. Conjugates were first characterized *in vitro*. Tissue-mimicking phantoms, imaging specimens of known geometric and material composition, were used to assess the sensitivity and depth penetration of the NIR-I/SWIR device, showing a minimum detectable volume of ∼0.9 mm^3^ and depth penetration up to 3 mm. *In vivo*, fluorescence imaging using the NIR-I/SWIR device showed a high tumor-to-background ratio (TBR) for both dyes, with anti–GD2-IR800 being significantly brighter than anti–GD2-IR12. Crucially, the system enabled higher TBR at SWIR wavelengths than at NIR-I wavelengths, verifying SWIR imaging enables high-contrast delineation of tumor margins. This work demonstrates that by combining the high specificity of anti-GD2 antibodies with the availability and translatability of existing NIR-I dyes, along with the advantages of SWIR in terms of depth and tumor signal-to-background ratio, GD2-targeted NIR-I/SWIR-guided surgery could improve the treatment of patients with neuroblastoma, warranting investigation in future clinical trials.

**Significance:**

Multispectral near-infrared I/shortwave infrared fluorescence imaging is a versatile system enabling high tumor-to-background signal for safer and more complete resection of pediatric tumors during surgery.

## Graphical Abstract

**Figure:**
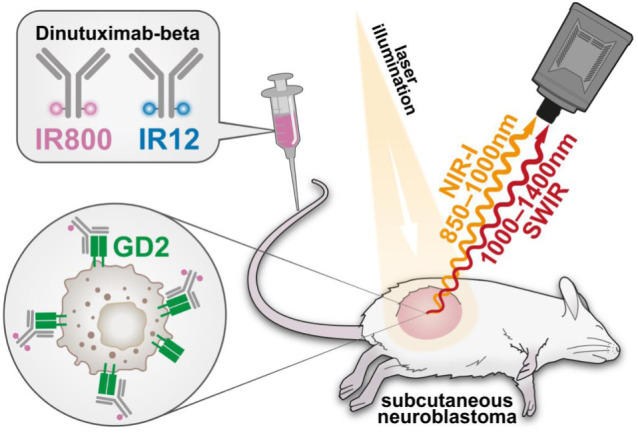

## Introduction

Neuroblastoma is the most common extracranial solid tumor in children, accounting for 8% to 10% of childhood malignancies and approximately 15% of cancer-related deaths in the pediatric population (1, 2). With at least one third of patients presenting with metastases at diagnosis, neuroblastoma is one of the most challenging diseases for pediatric oncologists and surgeons (3, 4). Despite the development of new therapeutic options, the prognosis of patients with high-risk neuroblastoma remains poor (4–6). Though there is some debate regarding the benefit of complete macroscopic excision, radical resection with no microscopic residual is considered the gold-standard in high-risk patients (7). Performing a radical excision is challenging due to neuroblastoma infiltrating the abdominal and thoracic cavities, major nerves and vessels, and its tight adhesions to the surrounding organs (3, 6, 8, 9).

Fluorescence-guided surgery (FGS) has the potential to address this challenge by providing a real-time visual map of tumors using fluorescent dyes. The detection of occult malignant tissue and positive tumor margins enables a radical resection to be performed whilst helping to preserve healthy surrounding structures (10, 11). Particularly, FGS achieves remarkable specificity when paired with tumor-specific fluorescently labeled probes. Though many of these tumor-specific agents have been developed and are currently under investigation in clinical trials in adult populations, none are yet available for use in pediatric oncology (12–14).

The specificity of FGS relies on the appropriate selection of tumor-specific targets (13). Regarding neuroblastoma, the disialoganglioside antigen GD2 represents a clinically relevant tumor-associated antigen due to its limited expression in normal tissue. Importantly, all neuroblastic tumors express GD2 receptors abundantly and ubiquitously regardless of tumor stage, with receptors persisting on the cell membrane after induction chemotherapy (15, 16). The development of GD2-targeted FGS is also facilitated by the availability of clinically approved monoclonal antibodies, such as chimeric anti-GD2 mAb ch14.18 (dinutuximab and dinutuximab-beta), which are the standard-of-care immunotherapies for patients with high-risk neuroblastoma (6).

Typically, FGS uses near-infrared (NIR) dyes emitting in the first biological window (NIR-I, 700–950 nm), where tissue shows diminished autofluorescence compared with visible light wavelengths, enabling higher target-to-background ratios. In addition, tissue is relatively transparent at those wavelengths due to decreased absorption and scattering from hemoglobin's, allowing deeper tissue penetration and visualization of sub-surface structures (12, 17). However, there is growing interest in the second biological window (NIR-II, 1000–1350), also known as the shortwave infrared region (SWIR), where autofluorescence, absorption and scattering are further reduced (18, 19). Despite the lack of clinically approved SWIR dyes, a growing selection of devices capable of measuring SWIR fluorescence is now available due to the decreased cost of the InGaAs sensors required to detect SWIR light (20). Moreover, recent studies show that several NIR-I dyes (such as IRDye800CW) have emission tails that stretch into the SWIR region. This characteristic allows them to be repurposed for SWIR FGS, exploiting the advantages of SWIR imaging whilst retaining the availability and translatability of NIR-I dyes (17, 21–23).

To investigate the potential of SWIR FGS, two such NIR-I dyes were conjugated to dinutuximab-beta in the current study: IRDye800CW, the most commonly used fluorophore conjugated to clinically approved monoclonal antibodies in clinical trials (24–26); and IR12, a NIR-I cyanine dye with reported benefits in the SWIR range (17, 18). A first-of-its-kind multispectral NIR-I/SWIR fluorescence imaging system was designed, constructed, and validated in a range of *in vitro* models. Finally, this novel system was combined with both conjugates and deployed in a preclinical study, enabling comparison of the probes in both the NIR-I and SWIR regions and evaluation of their potential for clinical translation.

## Materials and Methods

### Preparation of the antibody–dye conjugates

The chimeric monoclonal antibody dinutuximab-beta (Qarziba) was conjugated with IRDye800CW (LI-COR Biosciences) and IR12 (Nirmidas Biotech) with a dye to protein ratio of 1.5 (determined by UV-vis analysis). To reduce potential effects connected to the batch-to-batch variability of the products, each conjugate was produced in bulk and used for both *in vitro* and *in vivo* studies (Supplementary Fig. S1). The resulting two conjugates will be referred to as ‘anti–GD2-IR800’ and ‘anti–GD2-IR12’, respectively (protocol in Supplementary Methods).

To confirm conjugation, analytical high-performance liquid chromatography was performed on an Agilent Infinity 1260 quaternary pump system equipped with a 1260 Diode array (Agilent Technologies). Elution profiles were analyzed using Laura software v. 4.2.6.79 (Lablogic). Antibody–dye conjugates were analyzed on a bioZen SEC-2 column, 4.6 × 150 mm, 1.8 μm (Phenomenex) using 95% 0.1 mol/L phosphate buffer pH 6.8 and 5% isopropanol as mobile phase at a flow rate of 0.35 mL/min. The absorbance was recorded at the wavelengths of 280, 760, and 780 nm (Supplementary Fig. S1).

### Cell lines and spheroids formation

GD2-positive LAN-1 (neuroblastoma), KELLY (neuroblastoma) cell lines, and SUPT1 (human lymphoma) cell lines engineered to express GD2 were selected for this work. The SUP-T1 wild-type (WT) cell line was used as negative control. GD2 expression on the selected cell lines was confirmed and quantified by flow cytometry using the QuantiBRITE bead method (Supplementary Fig. S2). All cell lines were cultured in RPMI1640 medium (Merck Life Science) containing 10% FCS (Thermo Fisher Scientific), 100 IU/mL penicillin, and 100 μg/mL streptomycin (Merck Life Science) in a humidified incubator at 37°C, 5% CO_2_ and tested monthly for *Mycoplasma* species (LookOut Mycoplasma PCR Detection Kit, Merck). For spheroids formation, LAN-1 cells were grown to 80% confluency and detached with Trypsin-EDTA solution (Merck Life Science). One thousand cells were seeded and spun on a round bottom Ultra-Low Attachment 96 well (Corning Ltd.) in cell-specific medium. The plate was centrifugated at 300 rcf for 5 minutes and incubated at 37°C for 72 hours to allow cell aggregation. Cell lines were kindly provided by Prof. Anderson's group (University College London). Cells were used for *in vitro* and *in vivo* study up to a passage number of 25.

### Flow cytometry

LAN-1 and KELLY cell lines were grown to 80% confluency and detached with Trypsin-EDTA solution. The SUPT1 GD2-positive and GD2-negative were both grown in suspension. For each cell line, 1.5×10^6^ cells were harvested and divided into three FACS tubes. Initially, a viability staining was performed by resuspending the cells in 100 μL of PBS-viability dye (Zombie Green Fixable Viability Kit, BioLegend Inc.) and incubating at room temperature for 30 minutes. One tube per cell line was used as negative control (unstained). For the remaining tubes, primary staining was performed by adding 10 nmol/L of either anti–GD2-IR800 or anti–GD2-IR12 conjugates in 50 μL of PBS. Secondary staining was performed by adding anti-IgG Alexa Fluor 647 (BioLegend Inc.) at a 1:2 ratio with the primary antibody. Both times, the cells were incubated at 4°C for 30 minutes and then washed twice with 1 mL of PBS. Samples were acquired on an LSRII flow cytometer (BD Biosciences). The analysis was performed using FlowJo software version 10.6.2 (TreeStar). The signal-to-background (SBR) was calculated as the median fluorescence intensity of the stained cells divided by the median fluorescence intensity of the unstained cells.

### Fluorescence microscopy

LAN-1 cells were grown to 80% confluency and detached with Trypsin-EDTA solution. After counting, 8×10^5^ cells were plated into a 60-mm cell culture dish (Thermo Fisher Scientific) and stained with 100 nmol/L of either anti–GD2-IR800 or anti–GD2-IR12 and incubated for 1 hour at 37°C. LAN-1 spheroids were stained with 100 nmol/L of either anti–GD2-IR800 or anti–GD2-IR12 and incubated for 24 hours at 37°C. Both cells and spheroids were washed 3 times and stained with Hoechst 33342 (Thermo Scientific) for nuclear counterstaining. After the staining procedure, spheroids were transferred into a VWR35 mm confocal dish (VWR international LLC). Images of the adherent cells and Z-stack images of the spheroids were acquired using a Zeiss LSM 880 confocal laser scanning microscope (excitation: 633 nm at 100% laser power; detector range: 638–747 nm; Carl Zeiss) and then processed using Fiji software (27).

### Multispectral NIR-I/SWIR fluorescence imaging system

**Figure 1.**
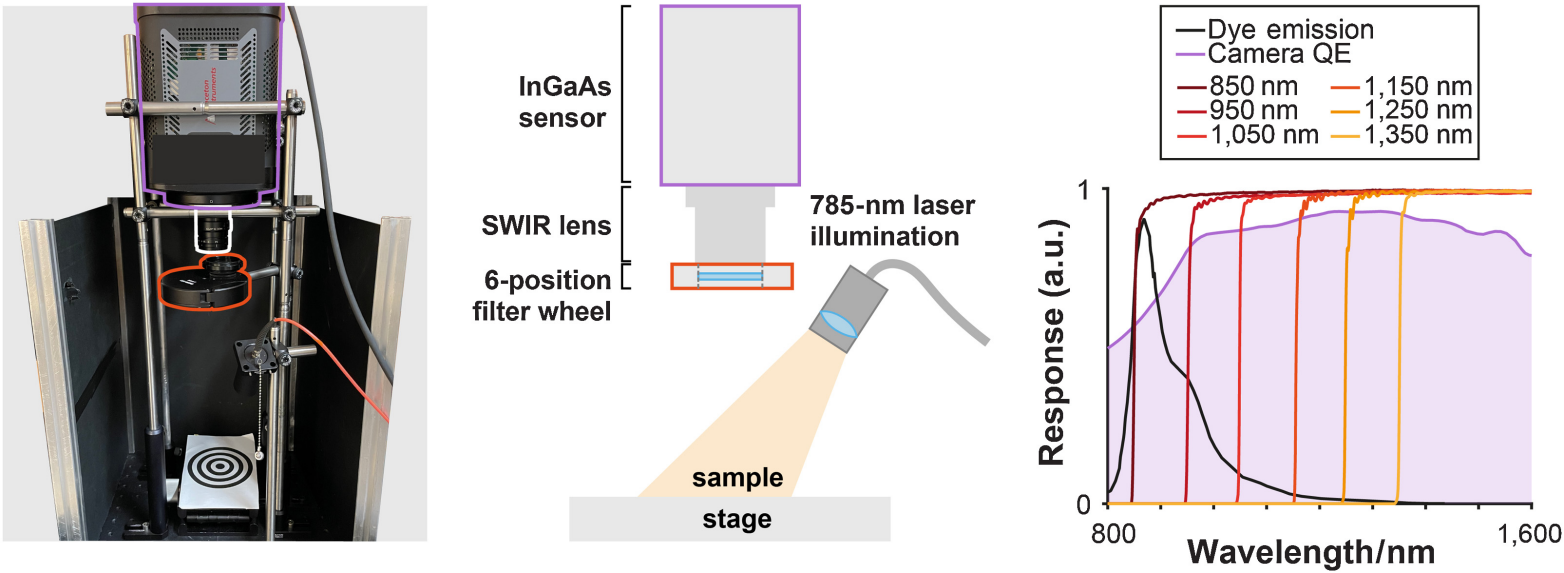
Multispectral NIR-I/SWIR fluorescence imaging device. Photograph of the custom multispectral NIR-I/SWIR fluorescence imaging system alongside its schematic representation. The spectral characteristics of the device are shown including the quantum efficiency (QE) of the sensor (purple line), a typical emission curve for cyanine dyes (black line), and the transmission for the long pass filters (orange lines).

A novel system was designed and constructed (Fig. 1). Briefly, tissue is illuminated by a 785-nm fiber-coupled laser (BWF-1–785/55371, B&W Tek) dispersed onto the sample using a ground glass diffuser (DG10–220-MD, Thorlabs). SWIR fluorescence emission from the sample is collected by a highly sensitive InGaAs camera (QE > 80% 950–1,600 nm, NIRvana 640, Teledyne Princeton Instruments) coupled to a SWIR lens (f = 16 mm, F/1.4, Navitar). Fluorescence light is sequentially filtered using a 6-position filter wheel (LTFW6, Thorlabs) through 6 long-pass filters with cut-off wavelengths of 850, 950, 1,050, 1,150, 1,250, and 1,350 nm (FELH series, Thorlabs). The system was mounted inside a light-tight enclosure to remove background light. The camera was cooled to −80°C to reduce thermal noise. Images were captured using LightField (Teledyne Princeton Instruments) and saved as 16-bit TIFs for analysis.

### *In vitro* cell imaging and analysis

To assess the sensitivity of the multispectral NIR-I/SWIR fluorescence imaging system, four sizes of cell pellets (2×10^6^; 1×10^6^; 5×10^5^, and 2.5×10^5^ in 200-μL microcentrifuge tubes (Fisher Scientific, United Kingdom) of four cell lines (LAN-1, KELLY, SUPT1 GD2-positive, and SUPT1-WT) were stained with 100 nmol/L of either anti–GD2-IR800 or anti–GD2-IR12. Imaging was performed using the multispectral NIR-I/SWIR fluorescence imaging system. Regions of interest (ROI) were drawn in the images around the stained (signal) and the control (background) pellets. Following dark subtraction and normalization for exposure, the mean fluorescence intensity (MFI) and SE in fluorescence intensity were calculated for each ROI. SBR was calculated by dividing the signal MFI by the mean background MFI for the cell line. Image processing and analysis were performed in MATLAB using custom scripts (MathWorks).

### *In vitro* tissue phantoms

To assess the ability of the multispectral NIR-I/SWIR fluorescence imaging system to image sub-surface fluorescence, LAN-1 and SUPT1-WT (used as negative control) cell pellets (2×10^6^ cells) were stained with 100 nmol/L of each conjugate. The stained pellets were placed in 200 μL microcentrifuge tubes and fixed horizontally on a Petri dish. A 2% emulsion intralipid phantom was then prepared through a 10-fold dilution of 20% emulsion intralipid (Sigma-Aldrich) with water to simulate realistic tissue scattering. The emulsion was poured into the Petri dish up to the pellet level, thus defining the 0-mm depth tissue phantom. Subsequently, more emulsion was added to completely submerge the tube with the cell pellet before imaging again. This process was performed to collect images at different depths. The fluid level was marked on the Petri dish with a red marker and subsequently measured with calipers at each depth.

### Xenograft model

LAN-1 cells (2×10^6^) resuspended in Matrigel (100 μL; Appleton Woods Ltd.) were injected subcutaneously on the right flank of 6- to 8-week-old athymic nude female mice (CD1-Foxn1nu, Charles River Laboratories). The LAN-1 cell line was established in 1977 from a bone marrow metastasis of a 2-year-old boy with stage IV neuroblastoma. Tumor growth was subsequently measured by caliper. Mice were intravenously injected with 100 μg (resuspended in 100 μL of PBS) of either of the two conjugates when the tumor was of adequate size (∼5×6 mm). Before any imaging procedures, the animals were anesthetized with 2.5% isoflurane for induction of anesthesia and 2% isoflurane for maintenance with a flow of 0.5 L/min.

### *In vivo* imaging of FGS probes using a commercially available clinical NIR-I imaging system

A small pilot study (*n* = 6) was performed to validate the tumor uptake of the conjugates *in vivo*. One mouse was euthanized because of the excessive tumor growth (humane end point), while another mouse did not show any tumor engraftment. The remaining four mice bearing subcutaneous LAN-1 tumors were intravenously injected with 100 μg (resuspended in 100 μL of PBS) of either anti–GD2-IR800 (*n* = 2) or anti–GD2-IR12 (*n* = 2). Mice were imaged using the EleVision IR platform (Medtronic Ltd., United Kingdom) every 24 hours after conjugate injection and culled at 96 hours. After euthanizing the mice, tumors were exposed, and resection was performed. Tumor excision was assessed under fluorescence for both mice using the open camera available on the EleVision IR platform. The camera was positioned at approximately 30 cm from the target, and the white light image with the fluorescence overlay was displayed in the main window. The laser boost, which determines the illumination power, was adjusted according to the observed emission intensities.

### Biological evaluation, biodistribution study, and *in vivo* SWIR imaging

**Figure 2.**
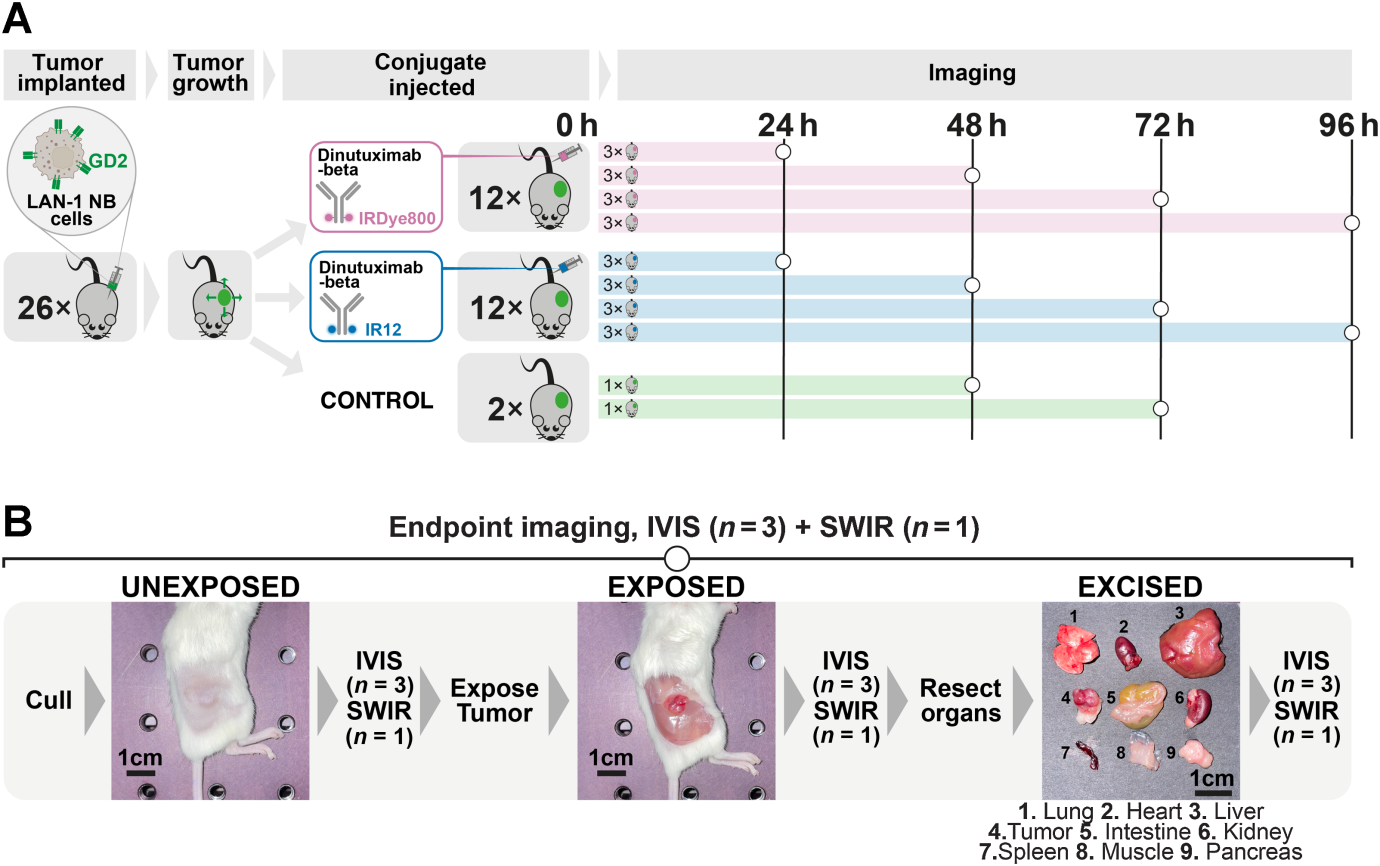
Overview of the *in vivo* preclinical study. **A,** Six to 8-week-old athymic nude female mice LAN-1 xenografts were injected with either anti–GD2-IR800 or anti–GD-IR12. The control mice were not injected. Mice were imaged at 24, 48, 72, and 96 hours using the IVIS Spectrum and the multispectral NIR-I/SWIR fluorescence imaging system. **B,** At each end time point, mice were euthanized and imaged using the IVIS (*n* = 3) and the multispectral NIR-I/SWIR fluorescence imaging system (*n* = 1). The unexposed tumors were imaged, followed by the exposed tumors and the excised organs of interest.

A biological evaluation of the two conjugates was performed using a preclinical imaging system (IVIS Spectrum In Vivo, PerkinElmer Inc., USA; excitation filter, center = 740 nm, bandwidth = 30 nm; emission filter, center = 790 nm, bandwidth = 20 nm). Mice bearing subcutaneous LAN-1 tumors were intravenously injected with 100 μg (resuspended in 100 μL of PBS) of either anti–GD2-IR800 (*n* = 12) or the anti–GD2-IR12 (*n* = 12; Fig. 2A). Two tumor-bearing mice, which were not injected with the conjugates, were used as a control and culled when the tumors reached a humane endpoint (48 and 72 hours). Fluorescence images were generated every 24 hours for four days following the injection of the two conjugates (Fig. 2A). At each time point, three mice from the anti–GD2-IR800 cohort and three mice from the anti–GD2-IR12 were euthanized, tumors were exposed, and selected organs were excised for biodistribution studies (Fig. 2B). In addition, at each time point, one mouse from each cohort was imaged using the novel multispectral NIR-I/SWIR fluorescence imaging device (Fig. 2B).

### *In vivo* fluorescence quantification

*In vivo* quantification of the fluorescent signal using the EleVision IR platform was performed using the built-in software. For images captured with the IVISSpectrum and the novel multispectral NIR-I/SWIR system, image processing and analysis were performed in MATLAB (MathWorks; details in Supplementary Materials).

### Multispectral analysis of NIR-I/SWIR fluorescence images

To investigate how fluorescence signals change with the wavelength in multispectral NIR-I/SWIR imaging, images captured using consecutive filters were subtracted to create an ‘image cube’, where each image contains a signal from a narrow band of wavelengths. For example, a 900-nm band image was produced by subtracting the image captured with a 950-nm long-pass filter from the image captured with an 850-nm long-pass filter, and thus contained signal from the range 850 to 950 nm. The final image cube is a 640 pixel × 512 pixels ×5 band cube with bands centered at 900, 1,000, 1,100, 1,200, and 1,300 nm and widths of 100 nm.

### Histologic analysis

Tissue samples were either fixed in formalin and paraffin-embedded or embedded in optimal cutting temperature (OCT) compound. Hematoxylin and eosin (H&E) and PHOX-2B [Recombinant anti-PHOX2B antibody (EPR14423) – C-terminal (ab183741), Abcam; dilution 1:500] staining were performed on sections from OCT samples (7 μmol/L–thick) by the Pathology Department at Great Ormond Street Hospital for Children (London, United Kingdom). Sections from the paraffin-embedded main tumor and tumor residual obtained from the *in vivo* pilot study were also imaged with the Odyssey CLx fluorescence flatbed scanning system (LI-COR Biosciences Inc.) with the following imaging settings (wavelength: 800 nm; resolution: 21 μm; quality: highest; intensity = 5). Briefly, sections were deparaffinized in xylene (6 minutes, twice) and left to dry in the air in a dark environment. After scanning the sections, H&E staining was performed. All slides were scanned using the NanoZoomer S360 digital slide scanner (Hamamatsu Photonics).

### Statistical analysis

Statistical analysis was performed using MATLAB (MathWorks) using Friedman tests or two-way ANOVA as defined in the text.

### Data availability

The data generated are available within the article and its Supplementary Data files. Raw data are available from the corresponding author upon request.

### Ethical statement

The project was developed according to established ethics approvals. All animal procedures were approved by Biological Services (Western Labs) at the University College London Great Ormond Street Institute of Child Health and were carried out following local and international regulations.

## Results

### Anti–GD2-IR800 and anti–GD2-IR12 specifically target neuroblastoma cell lines *in vitro*

**Figure 3.**
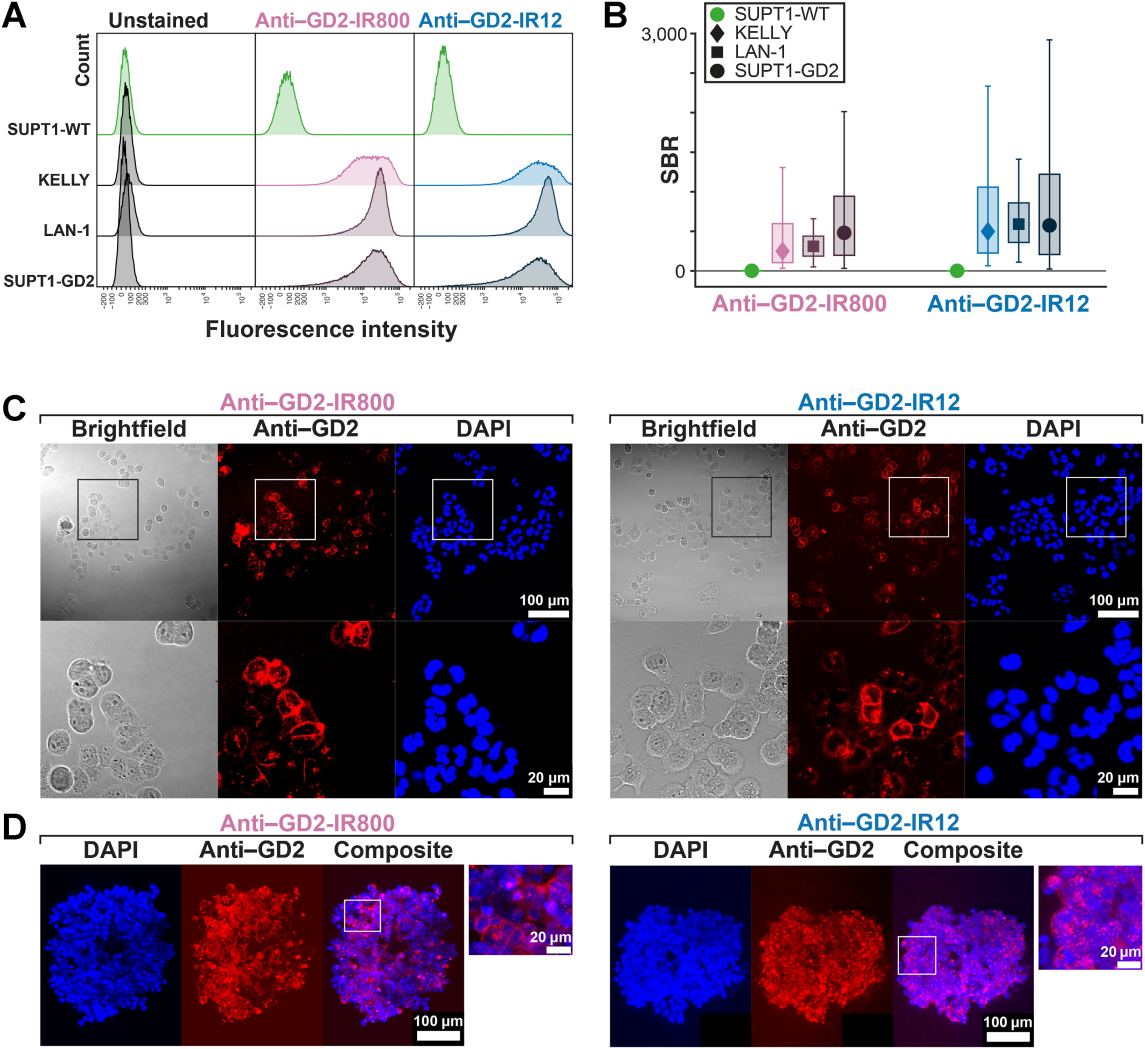
*In vitro* validation of anti–GD2-IR800 and anti–GD2-IR12. **A,** Binding specificity of anti–GD2-IR800 and anti–GD2-IR12 conjugates assessed by flow cytometry in three GD2-positive cell lines (KELLY, LAN-1, and SUPT1-GD2) and in the negative control (SUPT1-WT). **B,** Both conjugates show higher SBR (median stained fluorescence intensity/median unstained fluorescence intensity) for GD2-positive cell lines than for GD2-negative cell lines. Box represents interquartile range. Bars represent 5% to 95% range. Number of cells = 44,810–70,831. **C,** Fluorescence and brightfield images of the adherent cells 2 hours after staining with 100 nmol/L of either anti–GD2-IR800 or anti–GD2-IR12 (red). The nuclear counterstain was DAPI (blue). **D,** Representative section from the Z-stack acquisition of LAN-1 spheroids 24 hours after staining with 100 nmol/L of either anti–GD2-IR800 or anti–GD2-IR12 (red). The nuclear counterstain was DAPI (blue).

Specific binding of anti–GD2-IR800 and anti–GD2-IR12 was confirmed by flow cytometry analysis (Fig. 3A). Both conjugates showed higher SBR for GD2-positive cell lines compared with GD2-negative cell line (Fig. 3B). *In vitro* labeling of the adherent cell lines and LAN-1 spheroids showed a strong fluorescent cell surface signal with the diffusion of the conjugates up to the core of the spheroids (Fig. 3C and D). No relevant changes in tumor cell viability were observed after the staining *in vitro*.

**Figure 4.**
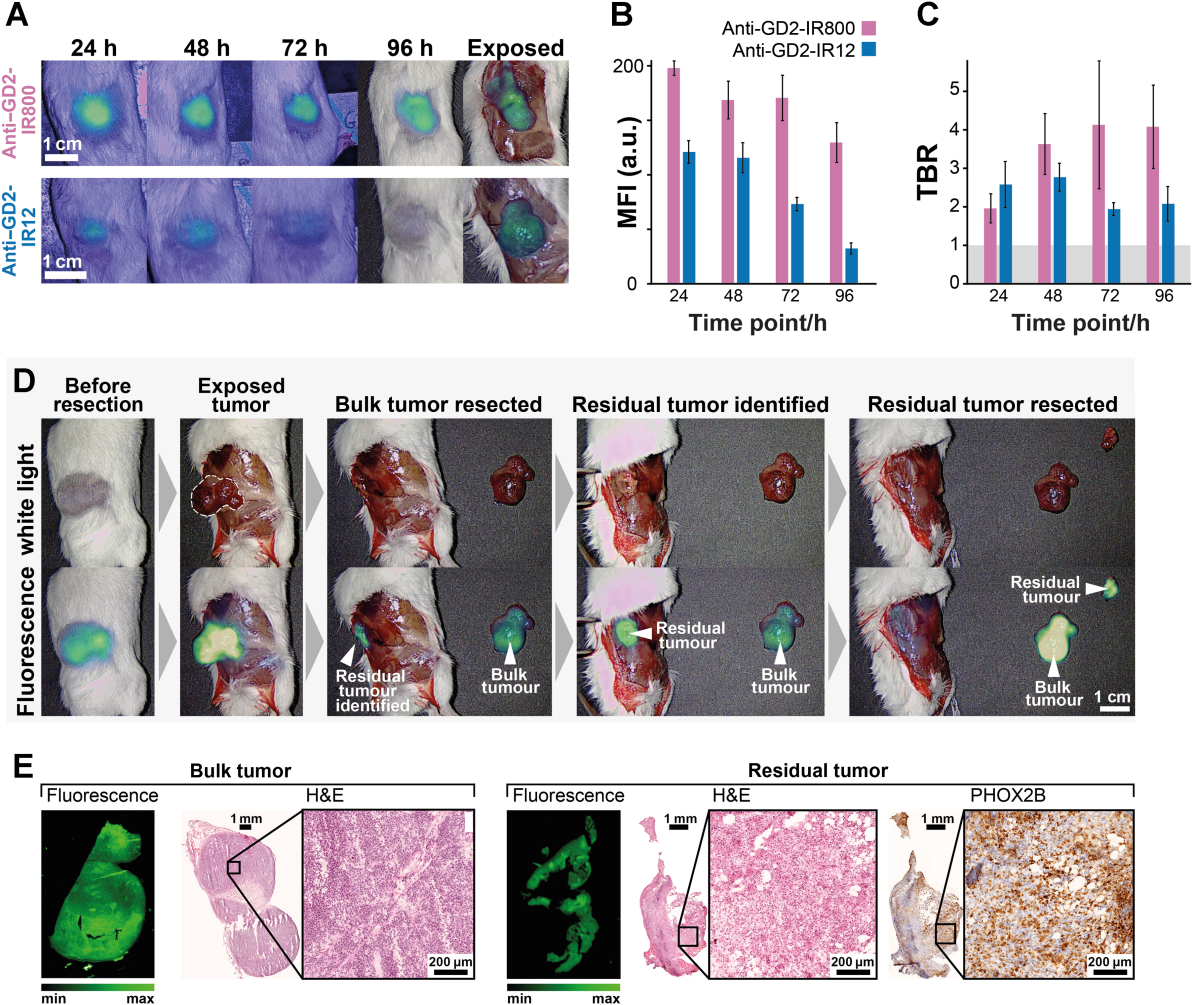
*In vivo* validation of tumor uptake of anti–GD2-IR800 and anti–GD2-IR12 using a commercially available clinical NIR-I imaging device. **A,** Comparison of fluorescent images of the unexposed tumors at different time points postinjection of either anti–GD2-IR800 or anti–GD2-IR12. The laser boost setting of the EleVision IR Platform was set to arbitrary units for 24, 48, 72, 96 hours and exposed anti–GD2-IR12 images, respectively. **B,** Bar chart showing MFI of the unexposed tumors post anti–GD2-IR800 and anti–GD2-IR12 injections. Quantification was performed using the EleVision software. **C,** Bar chart of the TBR. Error bars are calculated from the SD across 5 points manually selected in each ROI. Anti–GD2-IR800, *n* = 2 at all time points; anti–GD2-IR12, *n* = 2 at 24 hours, *n* = 1 at 48, 72, and 96 hours. **D,** Images showing stages of tumor excision after injection of anti–GD2-IR800 under white light observation and NIR-I fluorescence. A residual tumor (3 × 5 mm) was identified on fluorescence imaging and subsequently excised. **E,** Histopathologic evaluation of both the main tumor and residual tissue, confirming the presence of viable neuroblastoma.

Anti–GD2-IR800 and anti–GD2-IR12 allow high-contrast NIR-I tumor detection *in vivo* using a commercially available clinical NIR-I imaging system. To assess the conjugates’ uptake *in vivo*, four mice bearing subcutaneous neuroblastoma tumors were injected intravenously with 100 μg of either anti–GD2-IR800 (*n* = 2) or anti–GD2-IR12 (*n* = 2) and imaged using a clinical-grade NIR-I imaging device (EleVision IR Platform) up to 96 hours postinjection. Both conjugates allowed tumor detection at all time points (Fig. 4A), with 1.9 < TBR_IR800_ < 5.4 for anti–GD2-IR800 and 1.9 <TBR_IR12_ < 2.8 for anti–GD2-IR12. A decreasing trend of fluorescence intensity was observed for both conjugates over time (Fig. 4B and C), with anti–GD2-IR800 having a brighter signal compared with anti–GD2-IR12 (MFI_IR800_/MFI_IR12_ = 2.4 ± 1.2; *P* = 0.0455, Friedman test of dye effect; Fig. 4B and C). A higher ‘IR boost’ setting was required to acquire a satisfactory signal in anti–GD2-IR12 images compared with anti–GD2-IR800 images (typically, ‘IR boost’ was set at 6.0 arbitrary units for anti–GD2-IR12, and 1.0 for anti–GD2-IR800).

After euthanizing the mice, exposed tumors were excised under observation from the open field camera of the EleVision IR Platform. When FGS was performed to remove the tumor in one of the mice injected with anti–GD2-IR800 (Supplementary Video 1), the fluorescence signal allowed clear visualization of a positive margin in the surrounding tissue (Fig. 4D; Supplementary Fig S3). This area of infiltration, measuring 3 × 5 mm, was not detectable by naked eye or tactile palpation, and was confirmed as positive for neuroblastoma on histopathologic analysis, demonstrating the value of targeted fluorescence guidance in surgery (Fig. 4E). Sections from the paraffin-embedded main tumor and tumor's residual were imaged with the Odyssey CLx fluorescence flatbed scanning system and subsequentially stained with H&E to enable a direct correlation between the histology and the fluorescence signal (Fig. 4E). Neuroblastoma cells in the residual are more scattered compared with the solid bulk tumor and are mixed with fat tissue, perhaps explaining the absence of tactile and visual changes at the time of the surgery.

**Figure 5.**
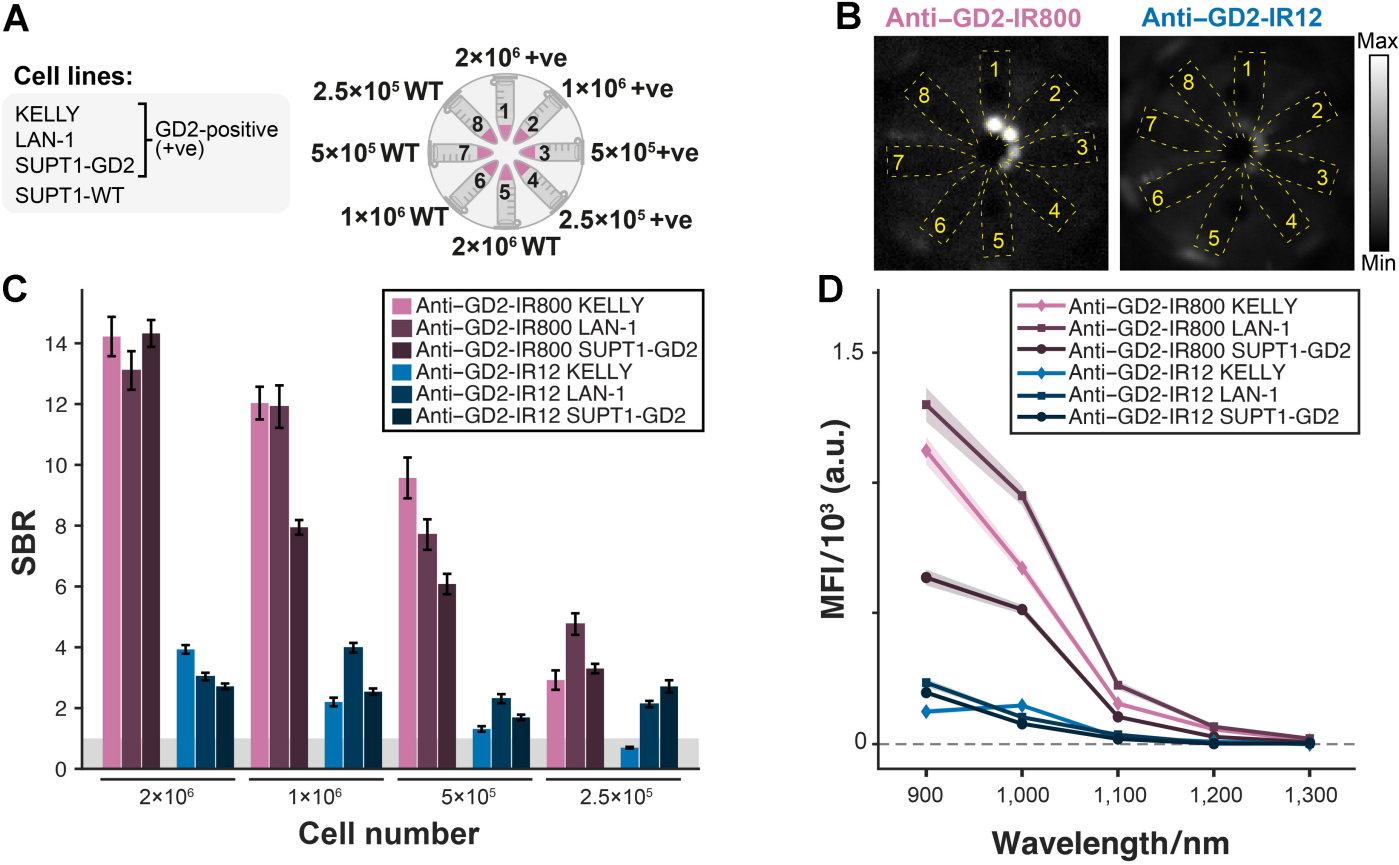
*In vitro* multispectral NIR-I/SWIR fluorescence imaging of anti–GD2-IR800 and anti–GD2-IR12 **A**. Schematic representation of selected cell lines (left) and number of cells used (right) to assess the camera sensitivity. Cells were stained with 100 nmol/L of anti–GD2-IR800 and anti–GD2-IR12 and prepared as pellets in microcentrifuge tubes [tubes 1–4, GD2-positive; tubes 5–8, SUPT1-WT (GD2-negative)]. **B,** Multispectral NIR-I/SWIR images were acquired. Images are shown for 1,050-nm LP filter LAN-1 cells (tubes 1–4) and SUPT1- WT cells (tubes 5–8) with anti–GD2-IR800 and anti–GD2-IR12 on the same fluorescence intensity scale. Yellow dotted lines show the approximate positions of Eppendorf tubes. **C,** The SBR was calculated as the MFI of each cell pellet divided by the MFI of the control cell pellets for the corresponding cell line and dye. Data are shown for the 850-nm long-pass filter. Error bars are derived from the SEs within the signal and background ROIs. **D,** MFI of anti–GD2-IR800 and anti–GD2-IR12 decrease towards longer wavelengths. The shaded region represents SE over pixels in ROI. Exposure time = 25 ms.

A novel multispectral NIR-I/SWIR fluorescence imaging platform enables sensitive detection of anti-GD2 labeled neuroblastoma cells *in vitro*. Because there are no commercially available SWIR fluorescence imaging platforms optimized for FGS, a first-of-its-kind multispectral NIR-I/SWIR fluorescence imaging device was constructed (see Methods for details). To assess the sensitivity of this new platform, three different GD2-positive cell lines and one GD2-negative control cell line were stained with the two anti-GD2 antibody–dye conjugates (Fig. 5A and B). The minimum detectable cell number was 2.5 × 10^5^ (corresponding to a volume of ∼0.9 mm^3^). Cells stained with anti–GD2-IR800 were consistently brighter than those stained with anti–GD2-IR12 (SBR_IR800_/SBR_IR12_ = 3.9±1.6, *P* = 5.3×10^−4^, Friedman test of dye effect; Fig. 5C). The emission of both dyes decreases towards higher wavelengths, as expected from the published emission spectra of the dyes (Fig. 5D; ref. 14).

### SWIR fluorescence imaging using anti–GD2-IR800 enables sub-surface detection of neuroblastoma cells *in vitro*

**Figure 6.**
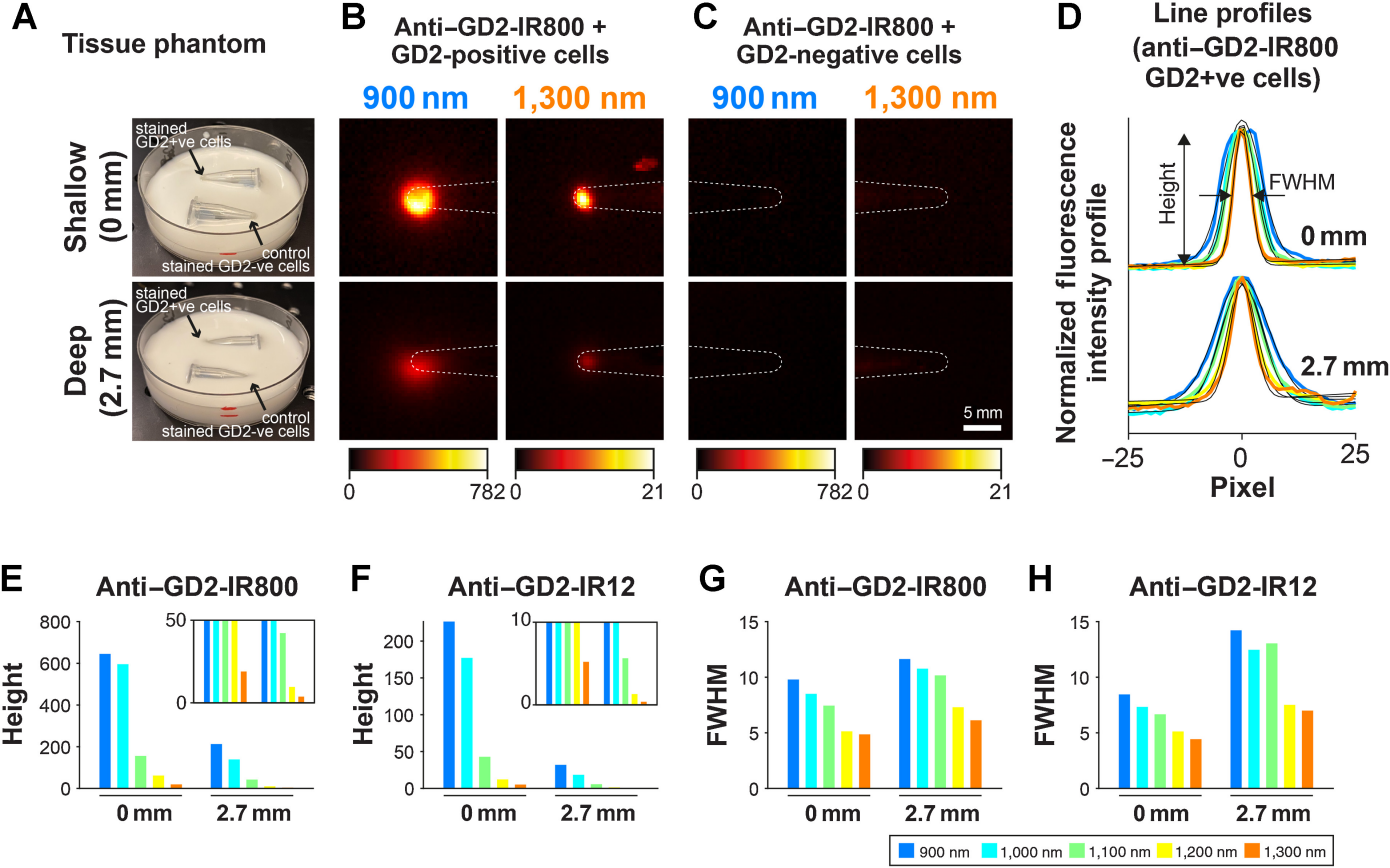
Imaging of stained cell pellets beneath tissue-mimicking material using multispectral NIR-I/SWIR fluorescence imaging. **A,** Microcentrifuges tubes containing pellets of 2 × 10^6^ GD2-positive cells (LAN-1) and GD2-negative cells (SUPT1-WT) stained with 100 nmol/L of anti–GD2-IR800 and covered with a 2% emulsion intralipid. The pellet is located at the tip of the tube. The fluid level is marked in red on the Petri dish. **B** and **C,** Multispectral NIR-I/SWIR fluorescence images were captured. Images show the anti–GD2-IR800–stained GD2-positive cells (LAN-1; **B**) and the anti–GD2-IR800–stained GD2-negative cells (SUPT1-WT; **C**). White dotted lines show the approximate location of the microcentrifuge tube. **D,** Line profiles across the fluorescence images were fitted with a Gaussian plus linear background to extract a height and FWHM. Fits shown as a black line. **E–G,** Bar graphs of the height (**E** and **F**) and the FWHM (**G** and **H**) versus depth for each wavelength band for GD2-positive (LAN-1) cells stained with anti–GD2-IR800 (**E** and **G**) and anti–GD2-IR12 (**F** and **H**).

To assess the feasibility of sub-surface SWIR fluorescence imaging, tissue-mimicking phantoms using cells pellets stained with the anti-GD2 conjugates, obscured beneath tissue scattering medium, were prepared and imaged with the multispectral NIR-I/SWIR device (Fig. 6A). The fluorescence signal appears as a blurred circle in the images due to the scattering of light in the phantom material (Fig. 6B and C). A Gaussian plus linear background was fitted to the line profiles of the signal, thus extracting a peak signal height and a full width half maximum (FWHM; Fig. 6D). As expected, the peak signal height decreases rapidly with the depth of lipid phantom in all bands (Fig. 6E and F). However, the FWHM of the signal is smaller for longer wavelengths, demonstrating the sub-surface imaging potential of SWIR fluorescence (Fig. 6G and H).

### Biological evaluation of anti–GD2-IR800 and anti–GD2-IR12 using a preclinical NIR-I imaging system *in vivo*

**Figure 7.**
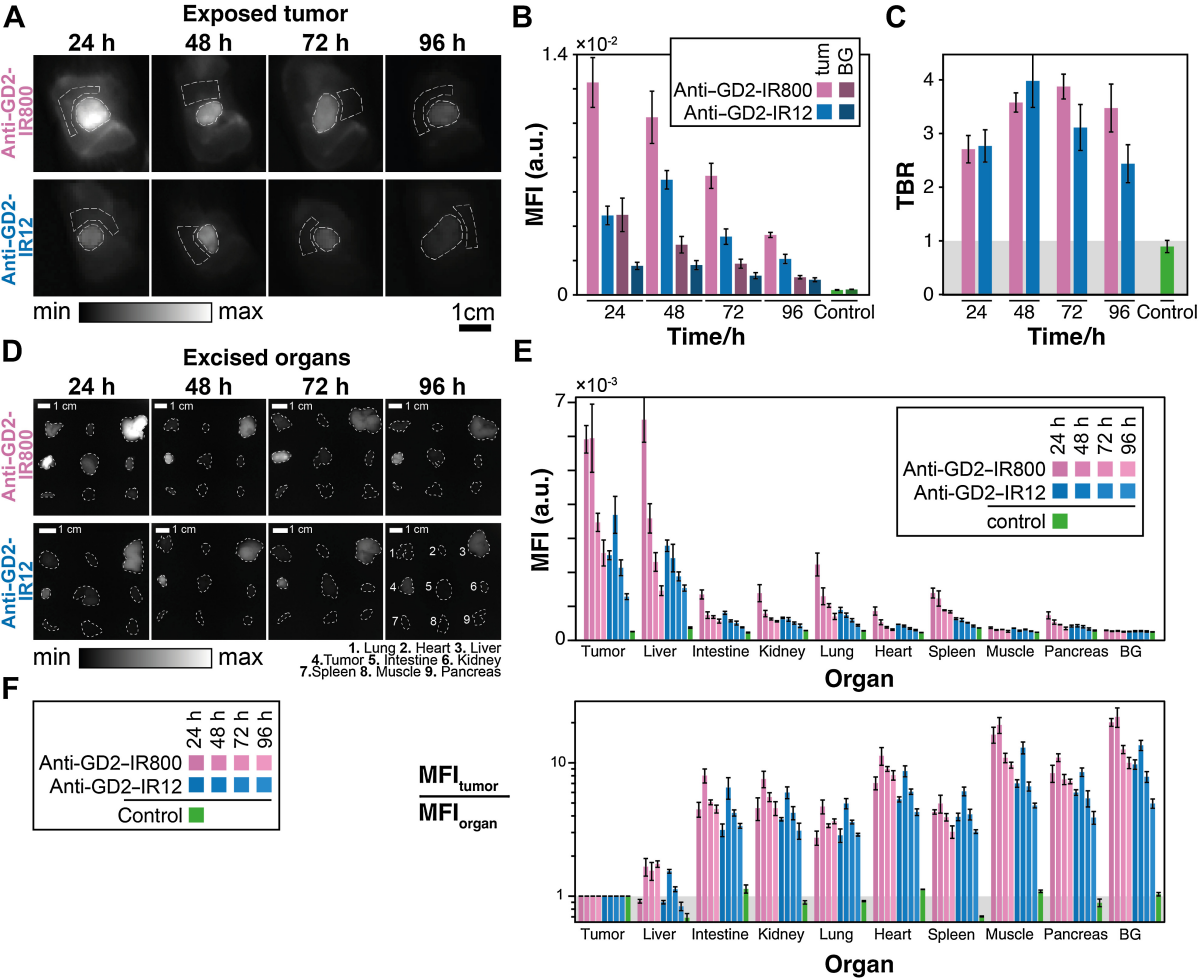
Biological evaluation of anti–GD2-IR800 and anti–GD2-IR12 using a preclinical NIR-I imaging system *in vivo*. **A,** IVIS Spectrum images of the exposed tumors at 24, 48, 72, and 96 hours postinjection of anti–GD2-IR800 (*n* = 3) and anti–GD2-IR12 (*n* = 3) in mice bearing subcutaneous LAN-1 neuroblastoma tumors. White dotted lines show the ROIs drawn to quantify tumor to background ratio. **B,** Graph bar showing fluorescence intensity of the tumor and the background at 24, 48, 72, and 96 hours post conjugates injection. **C,** Tumor-to-background ratio at 24, 48, 72, and 96 hours after the injection of the conjugates. Error bars represent the SE across individuals. **D,***Ex vivo* fluorescence images of the resected organs. The white dotted lines delineate the regions used to quantify the fluorescence signals. **E,** MFI for each organ at each time point and for the background card (BG). Fluorescence intensity decreases over time, with the anti–GD2-IR800 being consistently brighter than the anti–GD2-IR12 (MFI_IR800_/MFI_IR12_ = 1.90 ± 0.36 for tumor, *P* = 10^−20^, three-way ANOVA). Error bars represent the SE across individuals. **F,** MFI of tumor relative to MFI of each organ at each time point. Data for control mice is shown in green in the graphs **B, C, E,** and **F**.

Mice bearing subcutaneous LAN-1 neuroblastoma tumors were injected with either anti–GD2-IR800 or anti–GD2-IR12 and imaged at four time points (24, 48, 72, and 96 hours) using the IVISSpectrum imaging system (Fig. 7A; Supplementary Fig. S4). One anti–GD2-IR800 mouse was unintentionally not imaged in the exposed state at 24 hours. All animals were monitored and weighed daily, with no toxicity concern raised after the conjugate's injections. Fluorescence quantification confirmed preliminary data obtained with the clinical platform, showing a significant decrease of the MFI with time (*P* = 4.2×10^−6^, two-way ANOVA; Fig. 7B) and consistently brighter tumors with anti–GD2-IR800 compared with anti–GD2-IR12 (MFI_IR800_/MFI_IR12_ = 2.0±0.5, *P* = 2.7×10^−6^, two-way ANOVA; Fig. 7B). However, whilst the MFI decreased with time, the tumor remained detectable above background tissue at all time points for both conjugates [tumor-to-background ratio (TBR) > 1.5; Fig. 7C], with neither time nor dye, being a significant source of variance (*P* = 0.048 and *P* = 0.22 respectively, two-way ANOVA).

Upon excision and imaging for biodistribution (Fig. 7D; Supplementary Fig. S5), low MFI was observed in the heart, muscle and pancreas, while moderate MFI was observed in the intestine, kidney, lungs, and spleen. Both the tumor and the liver had high MFI (MFI_IR800, 24h_ = 5.9±0.4 and 6.5±0.7 ×10^−3^ respectively; Fig. 7E). Fluorescence signal decreased with time (*P* = 10^−28^, Friedman test for time effect), and anti–GD2-IR800 was significantly brighter than anti–GD2-IR12 (MFI_IR800_/MFI_IR12_ = 1.90±0.36 for tumor, *P* = 6×10^−17^, Friedman test of dye effect).

The tumor-to-organ ratio was calculated for each dye and at each timepoint (Fig. 7F). The tumor-to-liver ratio was highest at 48 and 72 hours postinjection, suggesting these might be the optimal times for FGS. The tumor-to-organ ratio for all other organs was large (2.7 < MFI_tumor_/MFI_organ_ < 19; Fig. 7F), so background fluorescence from these organs is not of concern.

### Multispectral anti-GD2 NIR-I/SWIR fluorescence imaging enables high-TBR delineation of tumors *in vivo*

At the time of the biological evaluation of anti–GD2-IR800 and anti–GD2-IR12, one mouse from each group was also imaged with the multispectral NIR-I/SWIR device at each timepoint (24, 48, 72, 96 hours). Longer exposure times were used at longer wavelengths to compensate for the exponential decay in fluorescence emission of the dyes (Supplementary Fig. S6).

A good agreement between the fluorescence intensity obtained with the NIR-I/SWIR device and the IVISSpectrum was found (linear fit, coefficient of determination R^2^ = 0.596; Supplementary Fig. S7). The tumor MFI after the administration of anti–GD2-IR800 was higher compared with anti–GD2-IR12 for both unexposed (MFI_IR800_/MFI_IR12_ = 2.9 ± 0.7; *P* = 0.013, Friedman test for dye effect; Supplementary Fig. S8A) and exposed tumors (MFI_IR800_/MFI_IR12_ = 3.0 ± 0.7, *P* = 0.013; Friedman test for dye effect; Supplementary Fig. S8B). Whilst the MFI decreased with time (*P* = 0.011, Friedman test for time effect), the tumor remained detectable above background tissue at all time points for both conjugates (2.3 < TBR < 4.1; Supplementary Fig. S8C and S8D), with neither time (unexposed, *P* = 0.24; exposed, *P* = 0.19, Friedman test for dye effect), nor conjugates (unexposed, TBR_IR800_/TBR_IR12_ = 0.97 ± 0.24; *P* = 0.32; Friedman test for dye effect; exposed, TBR_IR800_/TBR_IR12_ = 1.0 ± 0.2; *P* = 1.0; Friedman test for dye effect), being a significant source of variance, in line with the results from IVIS imaging.

**Figure 8.**
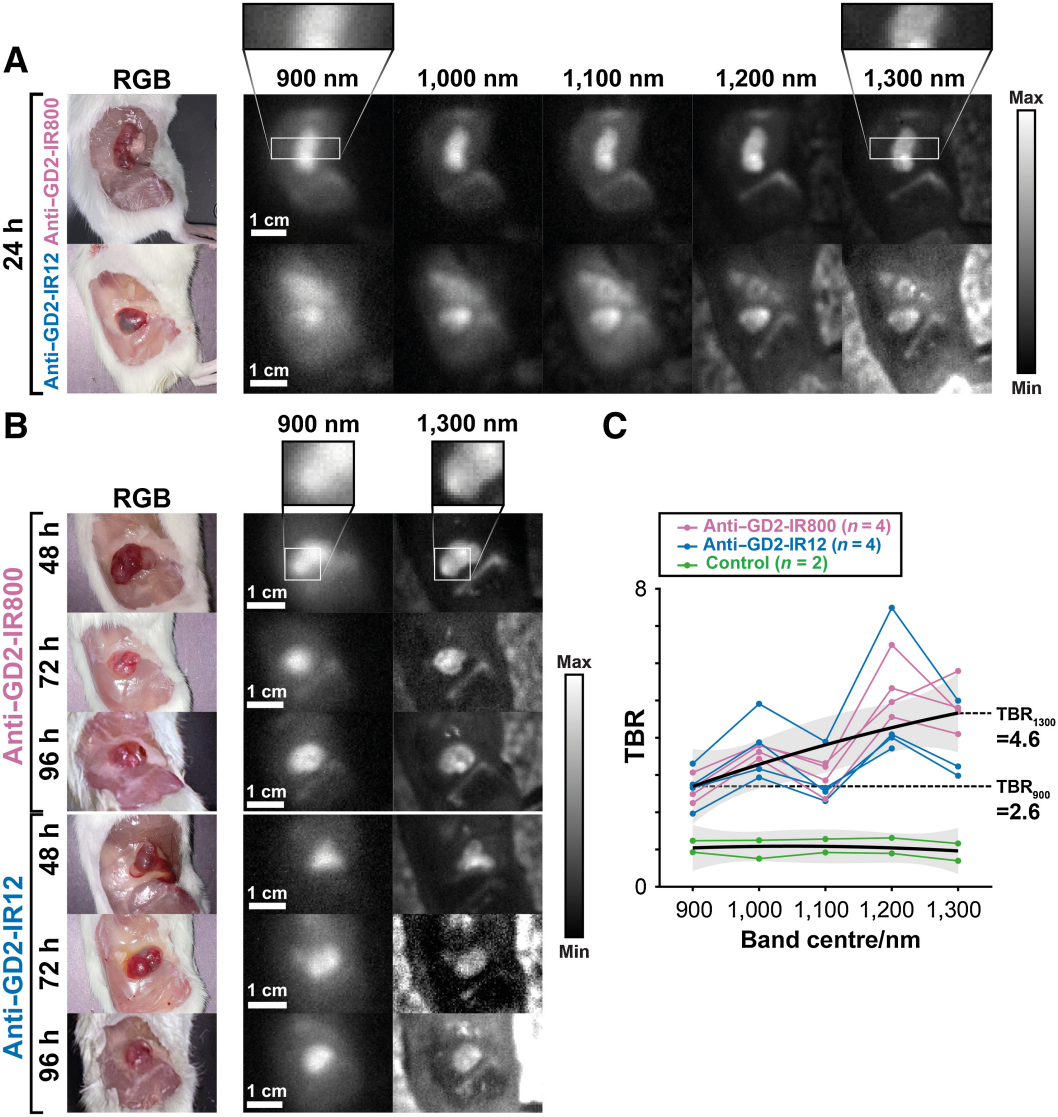
SWIR imaging achieves a higher TBR than NIR-I imaging. **A,** Normalized band images of exposed tumors at 24 hours post conjugates injection. Images show increased TBR at longer wavelengths. **B,** Normalized band images (900 and 1,300 nm only) 48, 72, and 96 hours after anti–GD2-IR800 and anti–GD2-IR12 administration. **C,** TBR versus wavelength band for each conjugate and control mice. Each line represents an individual (one individual per dye per time point). The TBR measured at 1,300 nm 72 hours post anti–GD2-IR12 injection was omitted as an outlier. The upper black line shows the fit of the combined anti–GD2-IR800 and anti–GD2-IR12 data. The lower black line shows the fit of the control data. The gray bands show the 95% confidence interval of the fit (simultaneous functional bounds).

Anti-GD2 SWIR fluorescence imaging achieves increased TBR and sharper margin definition than NIR-I fluorescence imaging *in vivo*. The multispectral NIR-I/SWIR fluorescence imaging device enabled the construction of band images, each representing light from a 100-nm acceptance band, allowing us to investigate the effects of imaging in different regions of the NIR-I and SWIR window (Fig. 8A and B). SWIR fluorescence (> 1,000 nm) showed a significantly higher TBR than NIR-I fluorescence (900 nm; mean TBR_900nm_ = 2.6 ± 0.4, mean TBR_1300nm_ = 4.6 ± 1.0, *P* = 3´10^−5^, Friedman test for wavelength effect; Fig. 8C). Specifically, anti–GD2-IR800 and anti–GD2-IR12 increased from a 2.2 < TBR_IR800, 900nm_ < 3.1 and 2.0 < TBR_IR12, 900nm_ < 3.3 in the NIR-I window, to a 4.1 < TBR_IR800, 1300nm_ < 5.8 and 3.0 < TBR_IR12, 1300nm_ < 5.0 in the SWIR. The increase in TBR provided a high-contrast delineation and an impressive high definition of the tumor margins.

Background signal from outside the mouse appears higher in the longer wavelength images due to the lower range of signal displayed. The positive SWIR fluorescence arising from the femur (Fig. 8A and B) was further investigated. Histopathologic analysis was negative for the presence of neuroblastoma cells, eliminating metastases as the source of the fluorescence. In addition, no fluorescence was detected in images of the femurs of the control mice (Supplementary Fig. S9), eliminating the possibility of autofluorescence as the source of this background signal. Some signal was observed when femurs were imaged using the IVISSpectrum system (Supplementary Fig. S10), suggesting the possibility of off-target conjugate binding. Further work is planned to investigate this off-target conjugate retention. In a pilot study conducted to evaluate this off-target effect by injecting anti–GD2-IRDy800 in nontumor-bearing mice, there was evident fluorescence in the femur at 24 hours (Supplementary Fig. S11).

## Discussion

Surgical resection of neuroblastoma is challenging due to the localization, heterogeneity, and aggressive behavior of the tumor, compounded with the lack of real-time tools able to distinguish malignant tissue from the surrounding healthy tissue (28). The introduction of FGS in neuroblastoma could transform its surgery by providing an objective, real-time tool to visualize the tumor and identify small residuals in locoregional or challenging areas. Our study demonstrates, for the first time, the potential for high-contrast NIR-I/SWIR fluorescence imaging both to achieve real-time visualization of the tumor and to provide a high-contrast definition of tumor margins and small residuals of disease.

The potential of anti–GD2-IR800 for intraoperative fluorescence imaging of neuroblastoma was recently evaluated by Wellens and colleagues, who showed that tumor xenografts, derived from pediatric tumor cell lines or patient-derived organoids, can be effectively detected *in vivo* using a real-time NIR-I intraoperative imaging system (14). The present study confirms these findings with NIR-I imaging of anti–GD2-IR800 and substantially expands the work to include additional cell lines, a second promising NIR-I probe (anti–GD2-IR12) and a first-of-its-kind NIR-I/SWIR imaging system. Crucially, the NIR-I/SWIR device was used to demonstrate that SWIR fluorescence imaging enables higher TBR, and superior depth penetration, compared with NIR-I imaging *in vivo*.

When imaging fluorescence below the surface, there is a complex trade-off between signal, sharpness (FWHM), wavelength and depth. To provide a complete picture, the present study measured both signal and sharpness versus depth. Signal decreases exponentially with depth in both NIR-I and SWIR, but because NIR-I dyes have intrinsically higher fluorescence emission at NIR-I wavelengths, NIR-I imaging achieves higher signals at all depths. Though the SWIR signal can be boosted by increasing exposure time, this must be balanced with the frame rate. In contrast, there is no way to compensate for the ‘blur’ caused by scattering at increased depths. Compared with NIR-I light, SWIR light has reduced scattering (20), so SWIR fluorescence imaging is valuable for sharper imaging at depth, albeit, with a lower overall signal. Intraoperatively, this has the potential to enable sharp visualization of small residuals, even when these are buried below the surface. For these reasons, we envisage translating SWIR imaging to the clinic as a valuable adjunct to NIR-I imaging, thus capitalizing on the advantages of both wavelength ranges to achieve better disease removal, particularly of tumors infiltrating vital organs and vessels where the plane of dissection is narrow and requires a sharp sub-surface image.

Unfortunately, SWIR fluorescent dyes are not available for human use, but the recently reported SWIR fluorescence tails of existing NIR-I dyes have the potential for use in SWIR fluorescence imaging. Two such dyes were investigated: IRDye800CW, which is commonly used in adult clinical trials (24–26); and IR12, a NIR-I cyanine dye with reported fluorescence in the SWIR range (17, 18). The off-peak SWIR fluorescence from anti–GD2-IR800 and anti–GD2-IR12 produced sufficient intensity to perform SWIR FGS at > 1,350 nm in subcutaneous neuroblastoma tumors. Notably, SWIR imaging achieved higher TBR compared with NIR-I imaging (TBR_1300nm_ = 4.6 ± 1.0 versus TBR_900nm_ = 2.6 ± 0.4). These results compare favorably to prior studies, where the off-peak SWIR fluorescence of anti–GD2-IR800 produced sharper images, with clear delineation of tumors and the cerebral cortex underneath the murine skull (23, 29, 30). Similarly to our findings, Zhu and colleagues showed that SWIR imaging using an anti–GD2-IR12 conjugate provided an increase in TBR compared with NIR-I imaging in squamous cell carcinoma xenograft models (22).

Higher MFI was observed for anti–GD2-IR800 compared with anti–GD2-IR12 throughout the study. This contrasts with the results obtained by Zhu and colleagues, who reported that IR12 is 2–3´ brighter than IRDye800CW (22). This discrepancy might partially be explained by the different absorption peaks of IRDye800CW (773 nm) and IR12 (767 nm) relative to the laser wavelength (785 nm, IRDye800CW at 75% peak absorption, IR12 at 61% peak absorption). In addition, the fluorescence intensity of IR12 could be reduced due to its fast hepatobiliary clearance route (over 80% in 24 hours; refs. 22, 31).

A nonhomogeneous distribution of tumor fluorescence was observed, particularly when moving into the SWIR wavelengths. This might be explained by the microscopic characteristics of the tumor, with areas of densely packed viable cells being associated with higher levels of fluorescence. Future work is planned to investigate the correlation between histologic features of the tumor and the fluorescence distribution.

When quantifying the effectiveness of fluorescence imaging data, particular importance must be given to TBR. It is still challenging to determine a threshold for sufficient contrast in humans because perceived TBR can vary depending on many factors, including the dynamic range of the imaging device, the observation conditions, and the choice of false-color representation in the images (12). In this regard, some studies stated that a TBR > 2.5 was sufficient to detect malignant tissue intraoperatively, while in others’ a TBR > 1.5 was sufficient for this purpose (32–34). Despite the differences in fluorescence intensity in our study, both anti—GD2-IR800 and anti–GD2-IR12 had a TBR > 2 at all time points (24–96 hours), which clearly distinguished the tumor from the surrounding tissues. Beyond this, we are exploring the use machine vision techniques to produce a binary classification map outlining the tumor based on fluorescence spectra across the NIR-SWIR range, thus making TBR obsolete and providing an objective, robust image segmentation (delineating tumor) to the surgeon.

The time between injection and imaging is important. Wellens and colleagues observed a decreasing MFI and increasing TBR with time, and thus defined 96-hours postinjection as the optimum imaging point (14). Our study also showed a decreasing MFI with time, but we observed maximum TBR at 72 and 48 hours postinjection for anti–GD2-IR800 and anti–GD2-IR12 respectively. Similarly to Wellens and colleagues, we found the liver to be the highest source of nontumor fluorescence, confirming hepatic clearance (14). The highest tumor-to-liver ratio was obtained at > 48 hours postinjection and 48 hours postinjection for anti–GD2-IR800 and anti–GD2-IR12 respectively. Overall, by considering the MFI, TBR, and tumor-to-liver ratios, we suggest 48-hours postinjection to be the optimum imaging time for FGS in NIR-I, but a dose-escalating clinical trial in humans is required to confirm the optimal dose of the conjugate (14).

An open challenge in FGS is that, despite the numerous NIR-I fluorescence imaging platforms available on the market, none can objectively quantify fluorescence intensity, and there is a lack of standardization of the images obtained. The standardization of fluorescence imaging devices for FGS, similar to that seen for established radiologic imaging methods, is an essential step forward that will strengthen the overall field, allowing quality control, performance benchmarking, and more comparable data between centers and diseases (35–38). Moreover, current commercial systems are optimized for imaging the few commercially available fluorescent dyes, the most ubiquitous being indocyanine green, the images produced are often processed, access to raw data is limited, and none have SWIR capabilities. To overcome this challenge, we designed and constructed our own multispectral NIR-I/SWIR fluorescence imaging device able to measure fluorescence emission in the range 850 to 1,350 nm.

Encouraged by the results presented in the current study, a clinical trial using FGS in neuroblastoma is recommended. Our findings support the translation of a multispectral NIR-I/SWIR fluorescence imaging system, like the one presented in our paper, to equip the surgeon with a versatile system for FGS.

Dinutuximab and dinutuximab-beta, alongside other commercial anti-GD2 therapeutic antibodies, have well-documented toxicity profiles at therapeutic doses. The antibody used in this study (chimeric 14.18 derived from a mouse monoclonal 14.18 in which the murine Fab domains are fused to human IgG1 Fc) has been in clinical use for around 20 years and is standard of care in neuroblastoma treatment both in North America and Europe. The incidence of human anti-chimera antibodies (HACA) is low as is the incidence of allergic reactions to antibody infusion. Because the antibody is a GMP-grade agent with an established safety profile, the route to clinical translation is likely to be relatively smooth, justifying its use in this study. Future clinical trials must be carefully designed to identify the effective minimal dose and the best administration protocol for surgical imaging (39).

This study has some limitations. Due to the long imaging sessions, the study used a small cohort of mice when assessing the NIR-I/SWIR device, which limited our ability to investigate biological variability between individuals. Among the optimizations planned for a ‘Generation II’ system is an automated filter wheel that will enable fast image acquisitions, allowing us to investigate biological variability between individuals in the future.

SWIR imaging required long exposure times (∼2,000 ms) compared with NIR-I imaging (∼50 ms) due to the low emission of the NIR-I dyes in this region. This could present a challenge for clinical SWIR imaging, where short exposure times would be required to achieve the higher frame rates needed for real-time imaging (>20 frames per second). Future work to realize a ‘Generation II’ combined NIR-I/SWIR system will optimize system illumination, the field of view, lenses, and filters to increase the frame rate for both NIR-I and SWIR imaging. We foresee the surgeon using real-time NIR-I imaging to visualize tumor boundaries and large residuals left in the surgical bed, whilst at the touch of a button, capturing sharp SWIR images of small cancer residuals at particularly challenging points in the surgery.

There are also limitations relating to the preclinical model used. First, although other fluorescently labeled probes do not show relevant side effects in humans, the use of the antibody–dye conjugate might lead to potential adverse reactions, that could not be assessed in our model. Second, the use of a subcutaneous tumor did not give information regarding the advantage of using FGS in a situation of organs and vascular encasements. Further studies, using immunocompetent mice and an orthotopic model of neuroblastoma, are needed. These will enable greater insight into the advantages of FGS *in vivo*, including assessment of the tumor bed for infiltrating residuals and serial bisections to determine the *in* vivo detection limit.

This study presents *in vivo* validation for anti–GD2-IR800 as a promising probe for FGS in neuroblastoma and introduces a novel targeted fluorescent probe, anti–GD2-IR12. Crucially, we demonstrated that both conjugates can be repurposed as SWIR fluorescent probes, supporting a straightforward translation of SWIR imaging techniques into clinical practice. By combining the high-specificity of anti-GD2 antibodies with the availability and translatability of existing NIR-I dyes, and the advantages of SWIR in terms of depth and tumor SBR, GD2-targeted NIR-I/SWIR FGS shows great prospects for improving neuroblastoma surgery.

## Supplementary Material

Supplementary Data Supplementary methods and figures

Supplementary Video Supplementary video showing fluorescence-guided resection of a subcutaneous model of Neuroblastoma injected with anti-GD2 fluorescence probes
