# Supplementary material for: Shortwave Infrared Imaging Enables High-Contrast Fluorescence-Guided Surgery in Neuroblastoma
Source: Cancer Res. 2023 Mar 19;83(12):2077–89. doi: 10.1158/0008-5472.CAN-22-2918 (PMC10267675; doi:10.1158/0008-5472.CAN-22-2918)
Supplement: Supplementary Data — Supplementary methods and figures [file can-22-2918_supplementary_data_suppsmsf1-sf11.docx]

**SUPPLEMENTARY MATERIALS**

**SUPPLEMENTARY METHODS**

**Preparation of the antibody-dye conjugates.** Initially, the buffer of Dinutuximab-beta (Qarziba, 4.5 mg/mL, UEUSA Pharma, Netherlands) was exchanged to 0.1 M Phosphate buffer (pH 8.65–8.75) using an amicon ultra centrifugal filter (50 kDa MWCO, Merck, UK). To produce an IRDye800CW conjugate having a dye-to-protein ratio of 1.5:1, Dinutuximab-beta (1.5 mg, 10 nmol) in 0.1 M phosphate buffer (pH 8.65–8.75; 9.3 mg/mL) was incubated with IRDye800CW-NHS (30 nmol, 10 mM in DMSO) (LI-COR Biosciences, USA) at room temperature for 1 hour. To produce an IR12 conjugate having a dye-to-protein ratio of 1.5:1, Dinutuximab-beta (1.5 mg, 10 nmol) was diluted with 50 mM borate buffer (pH 8) to a final concentration of 4 mg/mL. Meanwhile, IR12-NHS (30 nmol, 3.8 mM in DMSO) (Nirmidas biotech, USA) was incubated with 1-(3-dimethylaminopropyl)-3-ethylcarbodiimide hydrochloride (EDCI, Sigma Aldrich, UK) (1.2 mg) in 0.1 M MES buffer pH 5 at 37 °C for 15 mins. The dye mixture was added to the antibody solution. The reaction was carried out at room temperature for 2.5 hours. Both conjugates were purified by gel filtration using zeba spin desalting columns (40 kDa MWCO) (Thermo Fisher Scientific, UK) equilibrated in PBS. The concentration and the dye-to-protein ratio of each conjugate solution were determined by UV-VIS analysis using a Nanodrop 2000 spectrophotometer (Thermo Fisher Scientific, UK).

**SUPPLEMENTARY FIGURES**

**
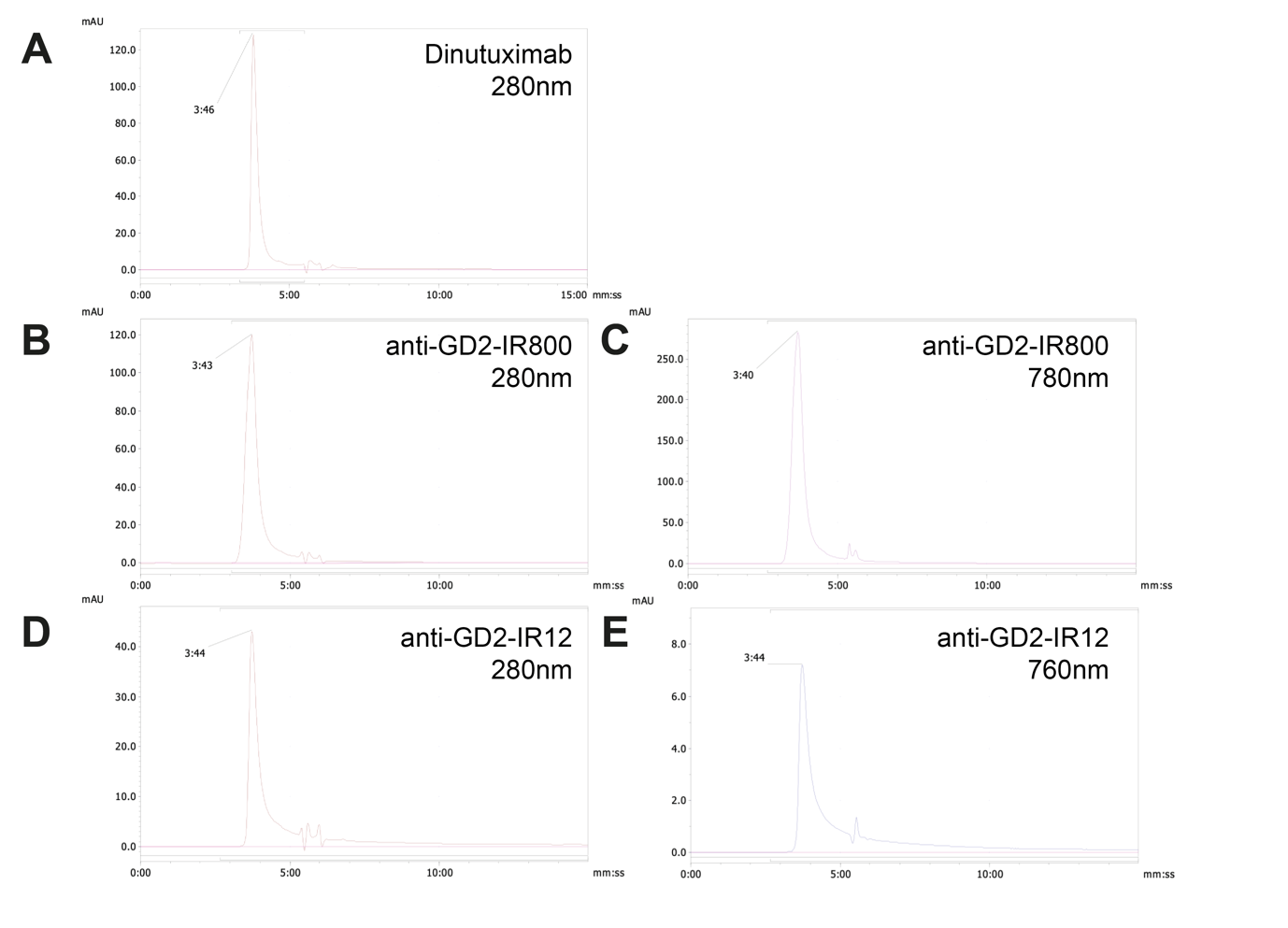
**

**Figure S1 *|* HPLC chromatograms.** **A.** Commercially available dinutuximab recorded at the wavelength of 280 nm. **B.** Anti-GD2-IR800 conjugate recorded at the wavelength of 280 nm **C.** Anti-GD2-IR800, 780 nm. **D.** Anti-GD2-IR12 conjugate recorded at the wavelength of 280 nm. **E.** Anti-GD2-IR12, 760 nm. The retention time (R_t_) is indicated as min:s. No high mass products (i.e., soluble aggregates) were observed. The low molecular weight impurities (<3%) eluting at approximately 5:30 min:s (**C** and **E**) could be free dye.


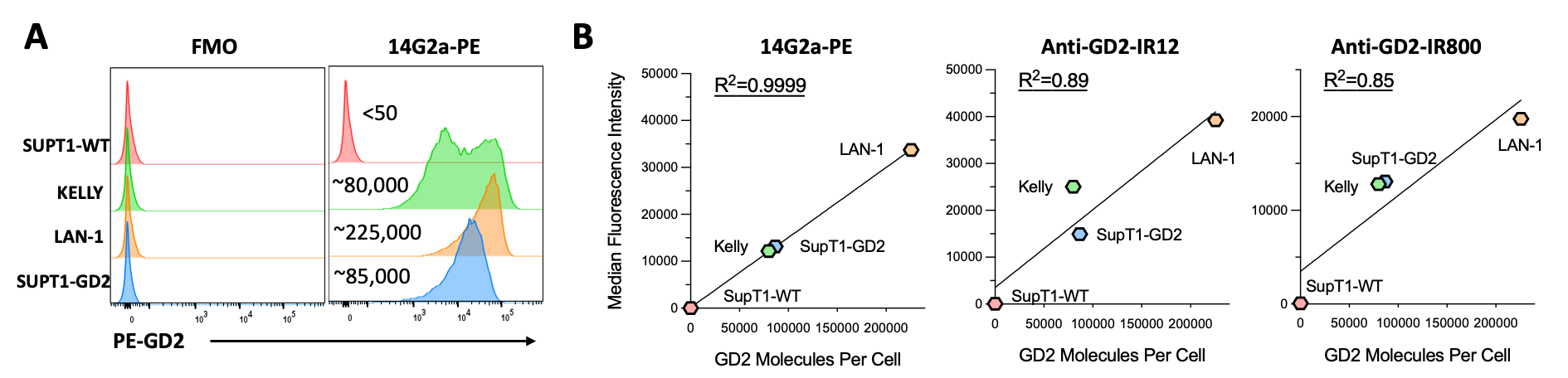


**Figure S2 *|* Figure S2 Comparison of fluorescence intensities of anti-GD2 probes and a commercially available anti-GD2 antibody. A.** Histograms show GD2 expression assessed by flow cytometry in three GD2 positive cell lines (KELLY, LAN-1, and SUPT1-GD2) and in the negative control (SUPT1-WT). Cells were stained with PE anti-human Ganglioside GD2 Antibody (clone 14G2a, 1:50 dilution). Numbers indicate GD molecules per cell line which was calculated using a PE fluorescence quantitation kit for flow cytometric (BD Biosciences™ Quantibrite™ Phycoerythrin Beads). **B.** Graphs showing the median fluorescence intensity of the commercial anti-GD2 antibody, anti-GD2-IR800, and anti-GD2-IR12 for each cell line (all three antibodies are 14G2a-derived binders). Black lines show a linear fit to data. R^2^ values show a good level of agreement between the commercial and the anti-GD2 probes.

**
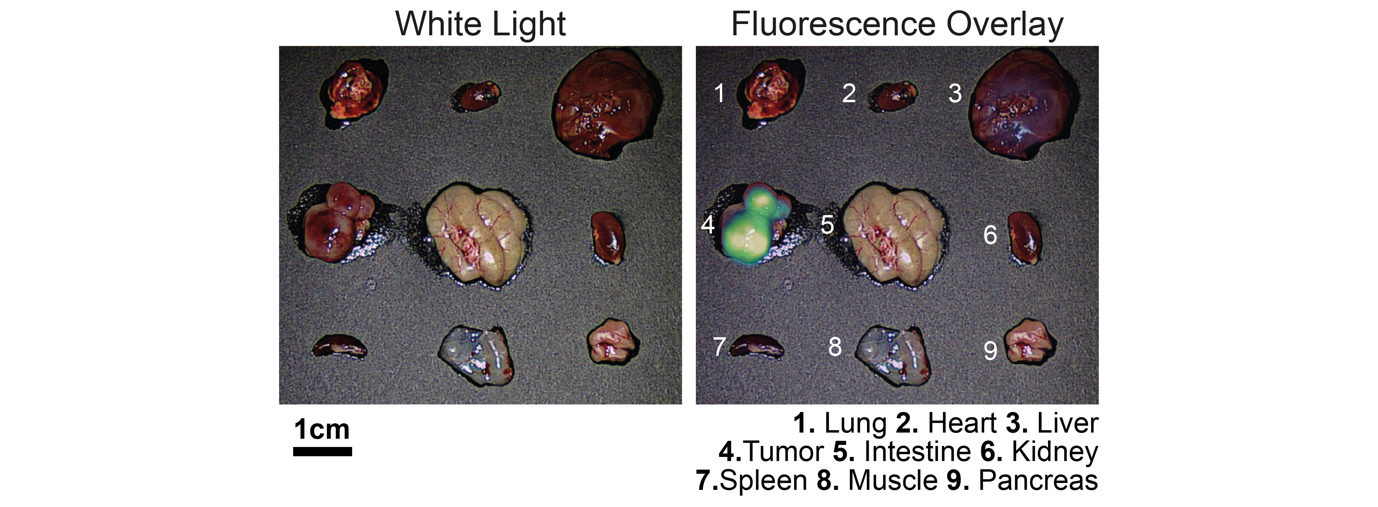
**

**Figure S3 *| Ex vivo* validation of tumor uptake of anti-GD2-IR800 using a commercially available clinical NIR-I imaging device.**


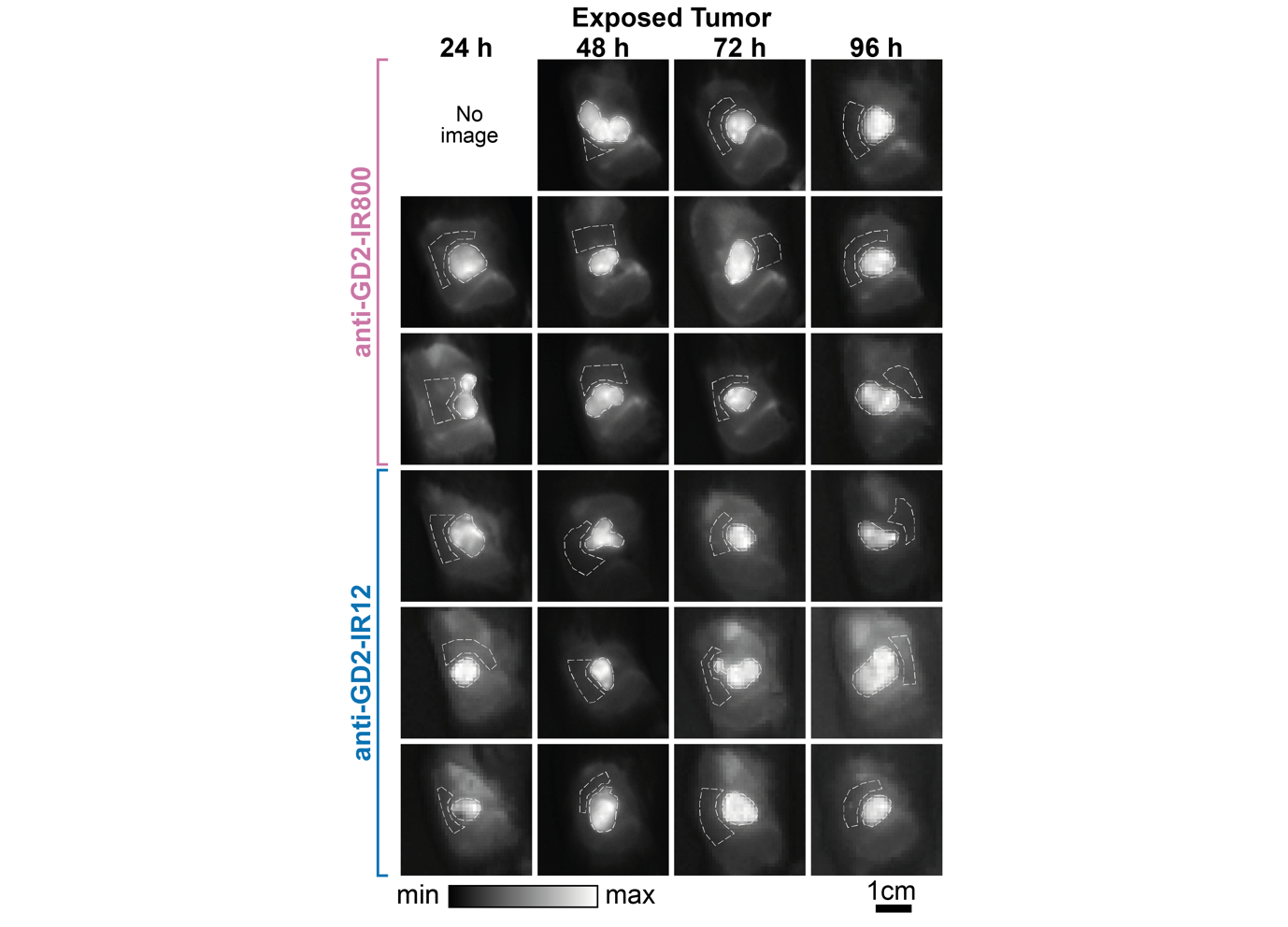


**Figure S4 *|* Fluorescence images of exposed tumors.** IVIS® Spectrum images of the exposed tumors at 24, 48, 72 and 96 h post-injection of anti-GD2-IR800 (n=3) and anti-GD2-IR12 (n=3) in mice bearing subcutaneous LAN-1 NB tumors. White dotted lines show the regions of interest drawn to quantify tumor to background ratio.


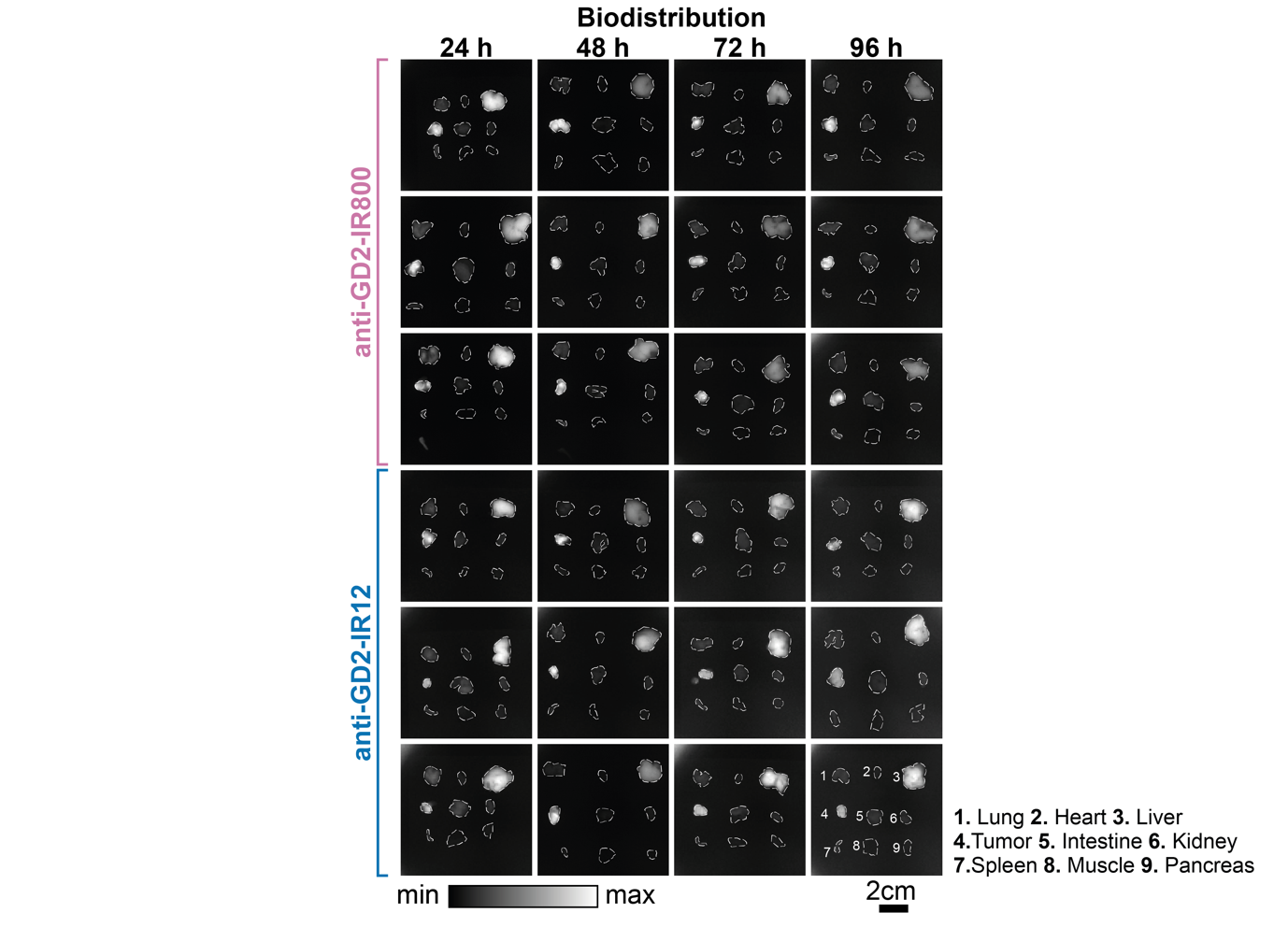


**Figure S5 *| Ex vivo* fluorescence images of the resected organs.** IVIS® Spectrum images of the resected tumors and organs at 24, 48, 72 and 96 h post-injection of anti-GD2-IR800 (n=3) and anti-GD2-IR12 (n=3) in mice bearing subcutaneous LAN-1 NB tumors. White dotted lines show the regions of interest drawn to quantify signal.


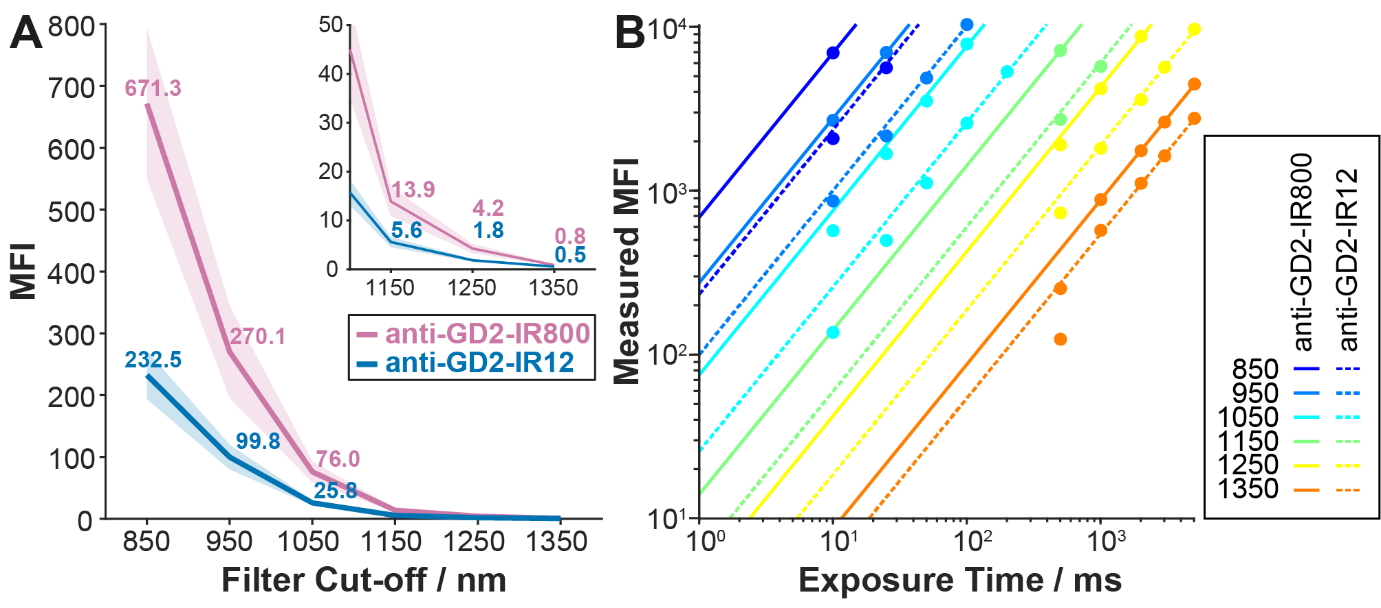


**Figure S6 *|* A comparison of the measured fluorescence intensity at different filter cut-off wavelengths and exposure times. A.** The exposure-normalized mean fluorescence intensity (MFI) exponentially decreases with filter cut-off wavelength (tumor region of 96 h mice). Shaded regions represent the standard deviation across the region of interest. **B.** The measured MFI increases linearly with exposure time, so longer exposure times were used for the longer wavelength filters. Solid and dotted lines show linear fit to data captured at a range of exposures from the 96 h anti-GD2-IR800 mouse and anti-GD2-IR12 mouse respectively.


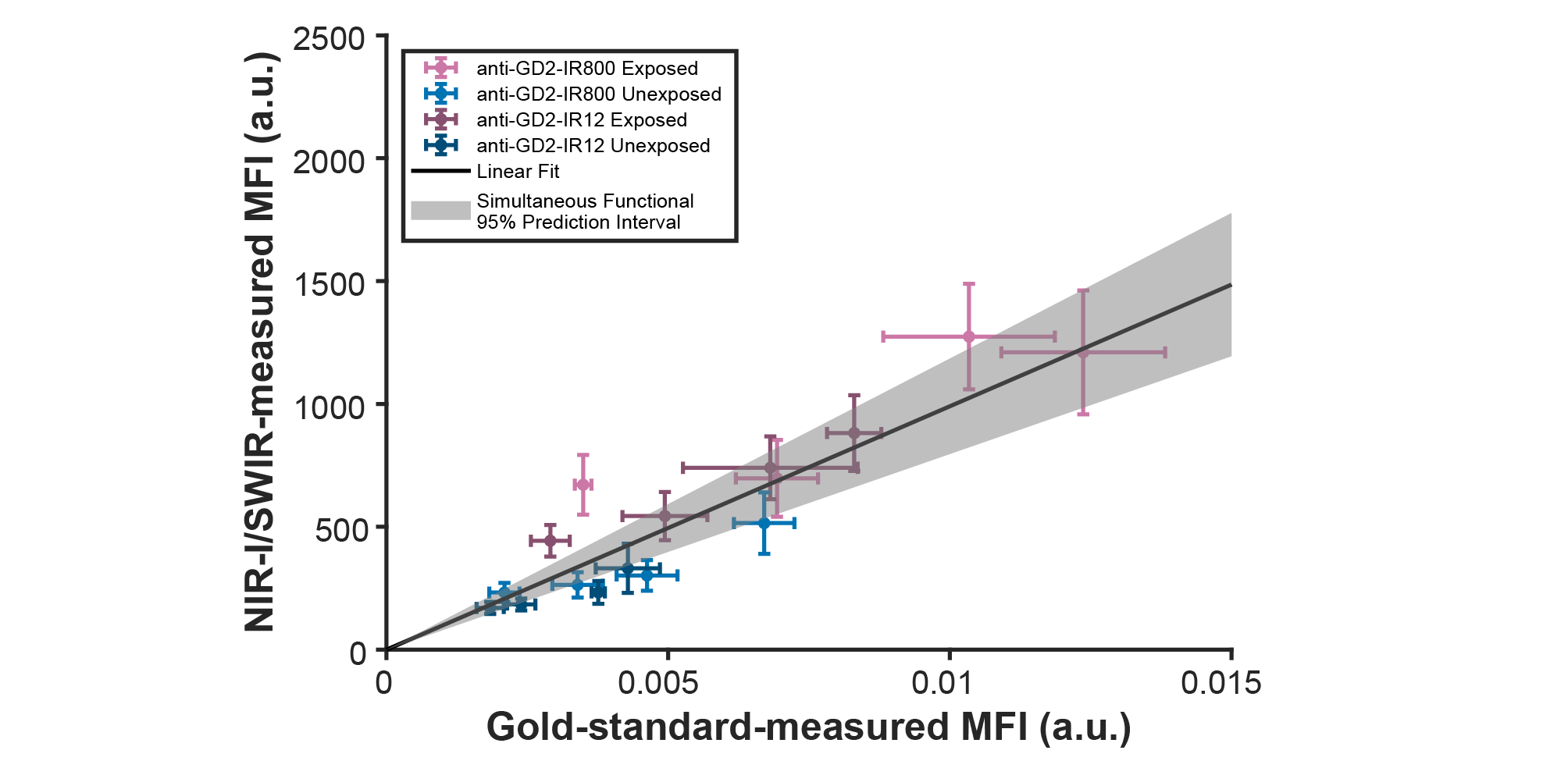


**Figure S7 *|* A comparison of mean fluorescence intensities (MFIs) measured by the gold-standard preclinical fluorescence imaging device and the multispectral NIR-I/SWIR fluorescence imaging device.** There was good agreement between fluorescence intensity obtained with the NIR-I/SWIR device and with the gold standard device (IVIS®Spectrum) (R^2^=0.596). Error bars from NIR-I/SWIR device are from standard deviation across ROIs. Error bars from the gold-standard device are from standard errors over individuals in the cohort (3 mice per timepoint). ‘Unexposed’ denotes data from images of the tumor with the tumour unexposed beneath the skin. ‘Exposed’ denotes data from images of the tumor with the tumor exposed.


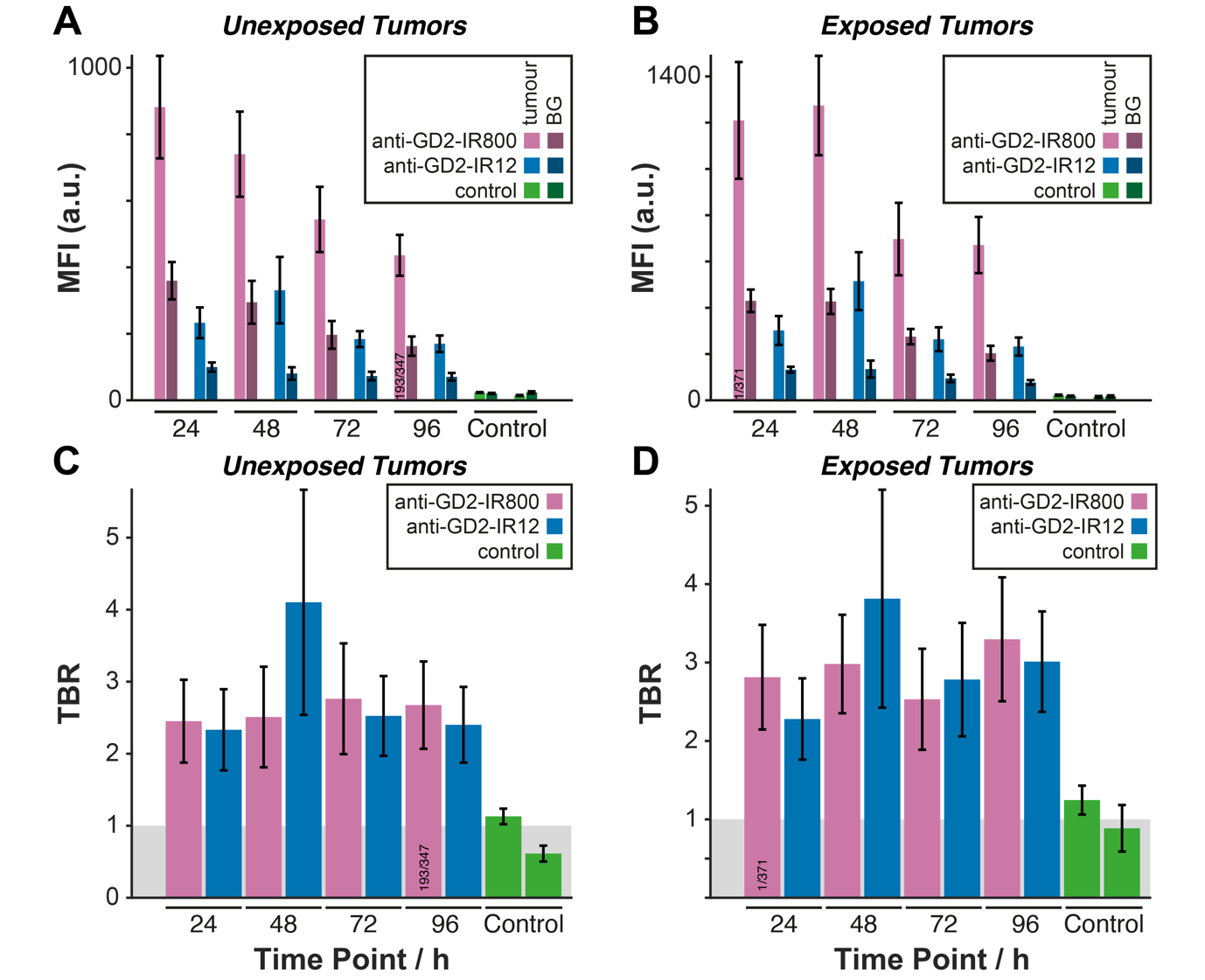


**Figure S8 *|* Multispectral NIR-I/SWIR fluorescence imaging enables high-TBR delineation of anti-GD2 labelled tumors *in vivo*.** Two tumor bearing mice were imaged with the multispectral NIR-I/SWIR device at each timepoint (24, 48, 72, 96 h), one from the anti-GD2-IR800 cohort and one from the anti-GD2-IR12 cohort. Tumors stained with anti-GD2-IR800 had a higher MFI than those stained with anti-GD2-IR12 for both **A.** unexposed (MFI_IR800_/MFI_IR12_ = 2.8 ± 0.7, p=0.013, Friedman test for dye effect) and **B.** exposed tumors (MFI_IR800_/MFI_IR12_ = 3.0 ± 0.7, p=0.013, Friedman test for dye effect). **C, D.** Whilst the MFI decreased with time (p=0.011, Friedman test for time effect), the tumor remained detectable above background tissue at all time points (2.3<TBR<4.1), with neither time (unexposed, p=0.19; exposed, p=0.19, Friedman test for dye effect) nor dye (unexposed, TBR_IR800_/TBR_IR12_ = 0.94 ± 0.22, p=1.0; exposed, TBR_IR800_/TBR_IR12_ = 1.0 ± 0.2, p=1.0, Friedman test for dye effect), being a significant source of variance, in line with the results from gold-standard imaging. Where saturated pixels were found in the images, numbers are added to the bars to represent saturated pixels / total pixels in ROI. Error bars represent the standard deviation over pixels within each ROI. The control bars are for 48 and 72 h mice left to right respectively.


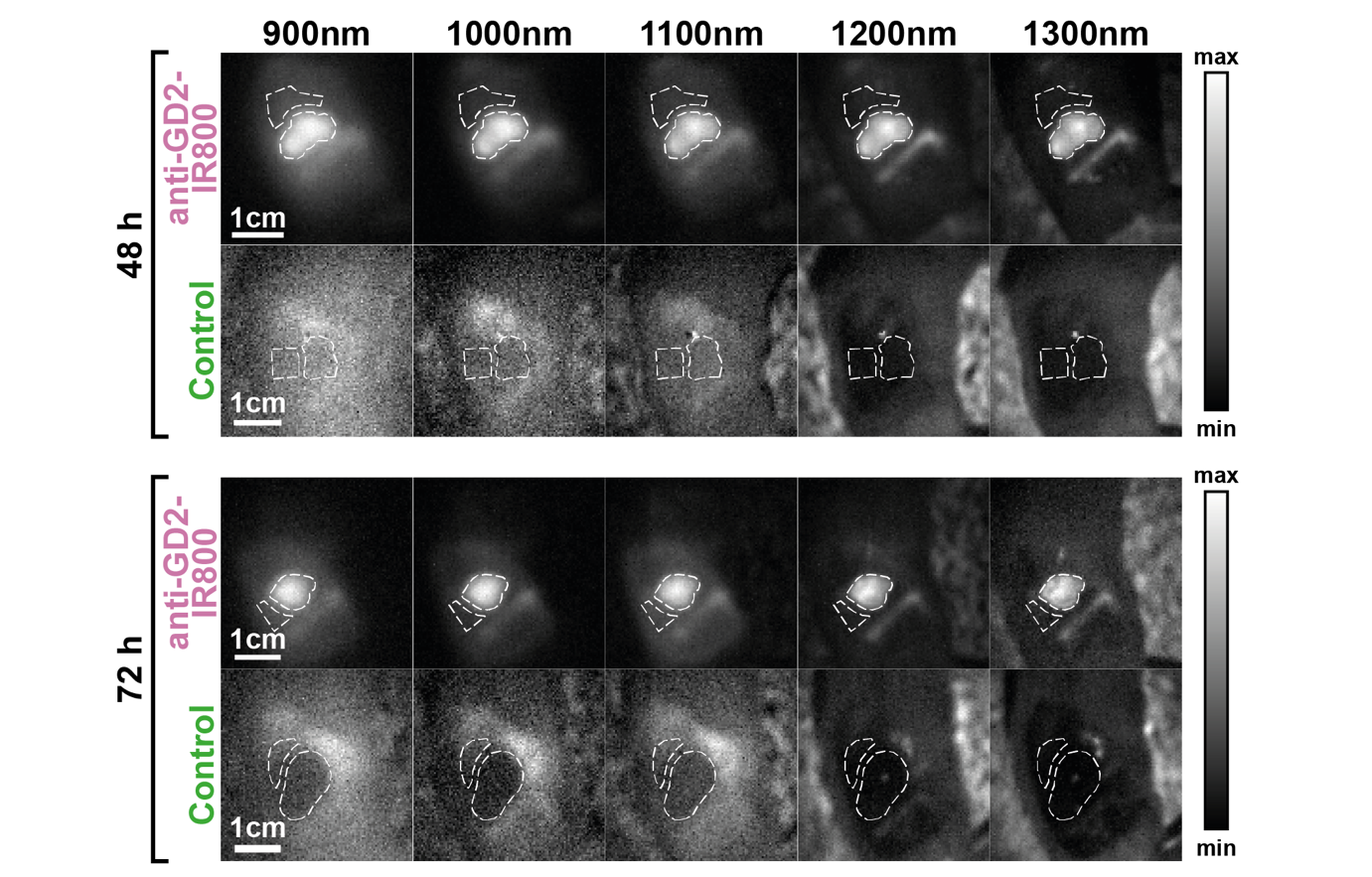


**Figure S9 | NIR-I/SWIR imaging control mice showing no visible fluorescence from the femur.** Normalized band images of exposed tumor at 48 and 72 hours. The dotted lines show the tumor and control (non-tumor tissue) regions. The femur, which is typically seen just below the outlined tumor region in the IR800 1300nm images, is not seen in any of the control images.


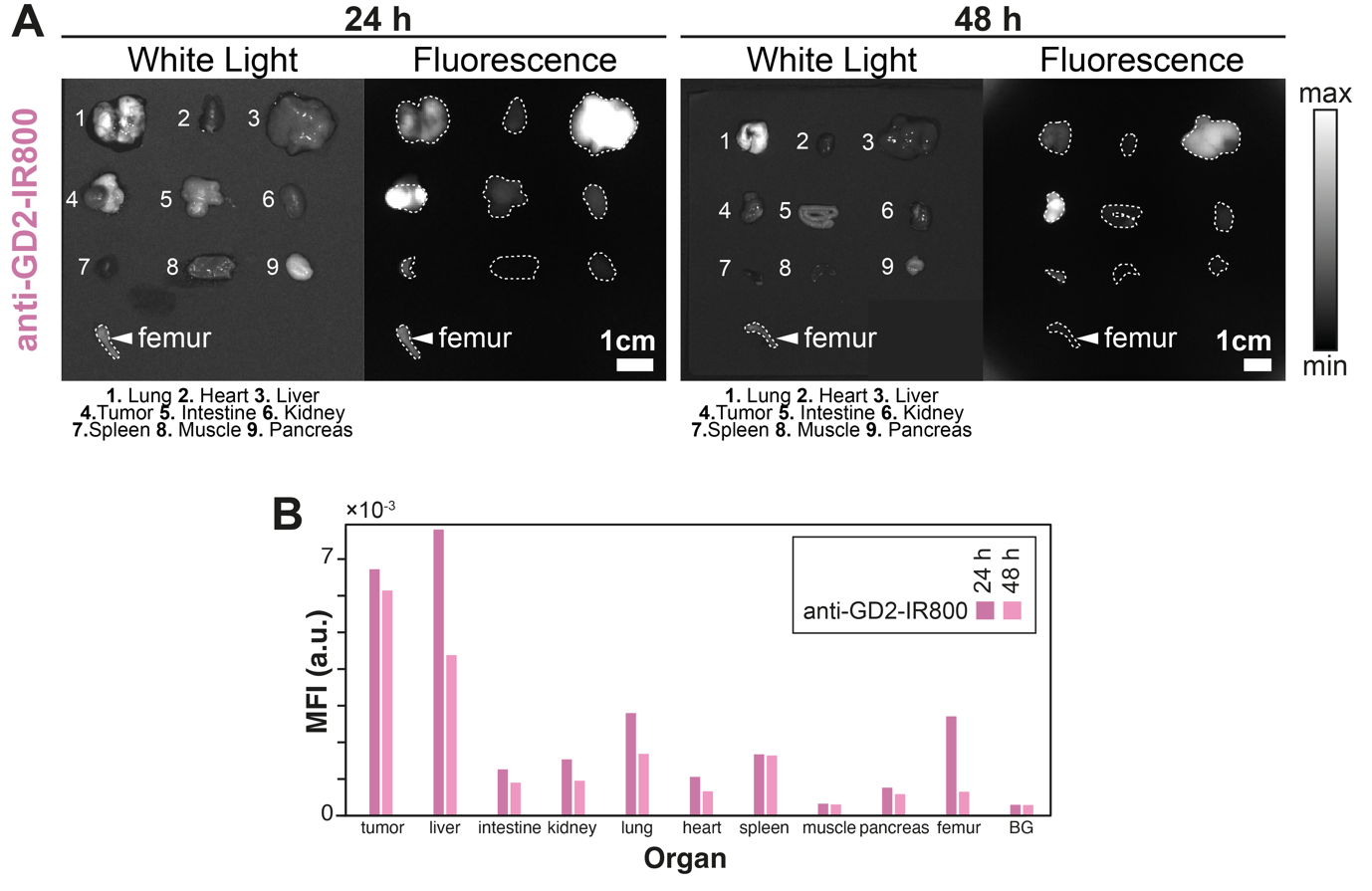


**Figure S10 *|* Femur imaged using the gold-standard preclinical fluorescence imaging device in two mice. A.** In two mice injected with anti-GD2-IR800, the femur was removed and imaged alongside the other organs. White dotted lines show the regions used to quantify fluorescence signals. **B.** Mean fluorescence intensity (MFI) for each organ at each time point and for the background card (BG).

**
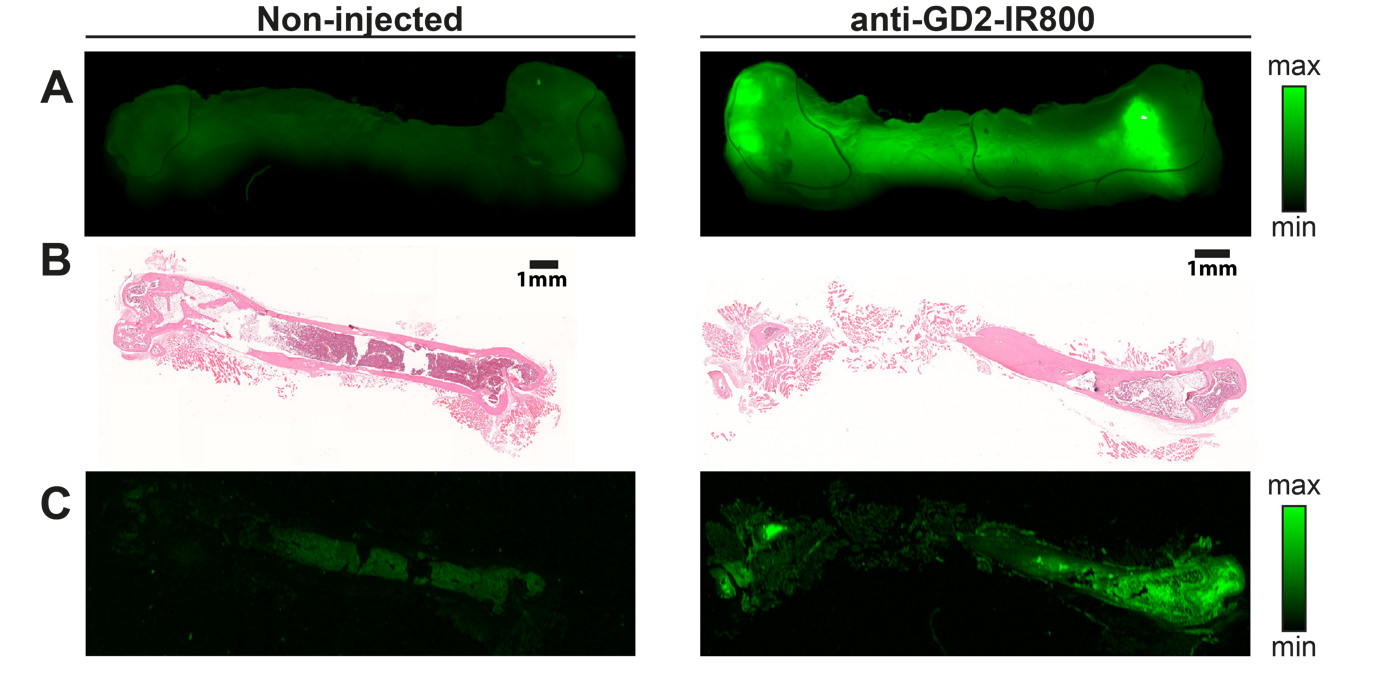
Figure S11 *|* Fluorescence imaging of *ex vivo* femur from non-tumor-bearing mice.**  Two non-tumour-bearing mice were used to investigate the off-target fluorescence in the femur, one injected with anti-GD2-IR800 and one non-injected. **A.** The femur was removed and imaged using the Odyssey® CLx fluorescence flatbed scanning system (LI-COR Biosciences Inc.) at 24 hours post-injection. **B, C.** Histopathological evaluation of the femur confirming the presence of off-target fluorescence in the femur.
